# Disruption of Epidermal Growth Factor Receptor but Not EGF Blocks Follicle Activation in Zebrafish Ovary

**DOI:** 10.3389/fcell.2021.750888

**Published:** 2022-01-17

**Authors:** Yanlong Song, Weiting Chen, Bo Zhu, Wei Ge

**Affiliations:** Department of Biomedical Sciences and Centre of Reproduction, Development and Aging (CRDA), Faculty of Health Sciences, University of Macau, Taipa, China

**Keywords:** epidermal growth factor EGF, EGF receptor EGFR, gonadal development, folliculogenesis, spermatogenesis, zebrafish

## Abstract

Folliculogenesis is controlled by intimate communications between oocytes and surrounding follicle cells. Epidermal growth factor (EGF/Egf) is an important paracrine/autocrine factor in vertebrate ovary, and it is well known for its stimulation of oocyte maturation. However, the role of EGF signaling through its receptor (EGFR/Egfr) in ovarian folliculogenesis is poorly understood, especially at early stages of follicle development. In this study, we created zebrafish mutants for Egf (*egf* ^*−/−*^) and Egfr (*egfra* ^*−/−*^ and *egfrb* ^*−/−*^) by CRISPR/Cas9 technique. Surprisingly, these mutants all survived well with little abnormality in growth and development. Spermatogenesis and folliculogenesis were both normal in *egf* ^*−/−*^ males and females. Their fecundity was comparable to that of the wildtype fish at 4 months post-fertilization (mpf); however, the fertilization rate of mutant eggs (*egf* ^*−/−*^) decreased significantly at 7 mpf. Interestingly, disruption of *egfra* (*egfra* ^*−/−*^) led to failed follicle activation with folliculogenesis being blocked at primary–secondary growth transition (PG-SG transition), leading to female infertility, whereas the mutant males remained fertile. The mutant ovary (*egfra* ^*−/−*^) showed abnormal expression of a substantial number of genes involved in oxidative metabolism, gene transcription, cytomembrane transport, steroid hormone biosynthesis, and immune response. The stunted PG oocytes in *egfra* ^*−/−*^ ovary eventually underwent degeneration after 6 months followed by sex reversal to males with functional testes. No abnormal phenotypes were found in the mutant of truncated form of EGFR (*egfrb*). In summary, our data revealed critical roles for EGFR signaling in early folliculogenesis, especially at the PG-SG transition or follicle activation.

## Introduction

Folliculogenesis is a dynamic process regulated by multiple endocrine and paracrine factors. In addition to pituitary gonadotropins, namely, follicle-stimulating hormone (FSH) and luteinizing hormone (LH), a variety of local ovarian growth factors also play important roles in controlling folliculogenesis in vertebrates, including epidermal growth factor (EGF) and its related peptides (Maruo et al., 1993; Schneider and Wolf, 2008; Tse and Ge, 2010).

EGF is a key member of the EGF family, which also includes transforming growth factor-alpha (TGFα), heparin-binding EGF (HB-EGF), amphiregulin (AREG), betacellulin (BTC), epiregulin (EPR), and epigen (EPGN). They can all bind and activate the common epidermal growth factor receptor (EGFR) (Harris et al., 2003). EGF family ligands and their common receptor EGFR are ubiquitously expressed in somatic tissues, and some family members are also expressed in the gonads at high levels. EGF, TGFα, and EGFR have been shown by immunohistochemistry to be produced in ovarian follicles of humans (Maruo et al., 1993; Bennett et al., 1996) and other mammalian species, such as the rat (Chabot et al., 1986) and hamster (Roy and Greenwald, 1990; Garnett et al., 2002). Further studies on the ovary have demonstrated that both EGF family members and EGFR are mostly expressed in somatic follicular cells, *viz*. granulosa and theca cells, of secondary, preovulatory and atretic follicles (Chabot et al., 1986), and the level of EGFR increases when follicles enter estrus phase (Conti et al., 2006). The expression of EGF and EGFR in the ovary is subjected to hormonal regulation. FSH stimulates EGFR expression during late folliculogenesis in mice (El-Hayek et al., 2014), and LH induces a rapid and transient expression of EGF family members and phosphorylation of EGFR in mouse follicle cells (Park et al., 2004). As a local ovarian growth factor, EGF is well known to stimulate oocyte maturation in a variety of mammalian and non-mammalian species (Dekel and Sherizly, 1985; Downs, 1989; Ding and Foxcroft, 1994; Lonergan et al., 1996; Rieger et al., 1998; Smitz et al., 1998; Pang and Ge, 2002). Further studies have shown that EGF-related growth factors play critical roles in mediating LH signaling in the follicle (Park et al., 2004), and EGF/EGFR signaling induces proliferation of granulosa/theca cells and enhances steroid hormone production (Yoshimura and Tamura, 1988; Hernandez and Bahr, 2003). Blocking EGFR pathway eliminated FSH-stimulated aromatase activity and estrogen production (Hsueh et al., 1981).

The functional importance of EGF family in mammals has been studied by the loss-of-function approach in the mouse. TGFα null mouse is healthy and fertile, except minor abnormalities in skin architecture and hair development (Mann et al., 1993). Targeted inactivation of EGF and AREG genes severely stunts ductal outgrowth in mouse mammary glands (Luetteke et al., 1999). Interestingly, all single ligand knockout mice are fertile and capable of producing live offspring (Mann et al., 1993; Luetteke et al., 1999). Disruption of EGFR gene in the mouse affects epithelial proliferation and differentiation to different degrees depending on the genetic background of the mouse strains used (Miettinen et al., 1995; Sibilia and Wagner, 1995; Threadgill et al., 1995). Since the EGFR null mice show pre-implantation or post-natal lethality (Sibilia and Wagner, 1995; Threadgill et al., 1995), its role in gametogenesis can hardly be investigated in the mouse model. Nevertheless, the meiotic resumption of oocytes seemed to be impaired in mutant mice with minimal EGFR kinase activity (Hsieh et al., 2007). Ovarian granulosa cell-specific knockout of EGFR showed slightly impaired fertility (Hsieh et al., 2011). Despite these studies on EGF/EGFR signaling in the ovary, its exact function in oogenesis still remains elusive. Although null models for EGF ligands and EGFR are available, most studies have focused on their roles in embryonic development and premature lethality, not reproduction (Miettinen et al., 1995; Sibilia and Wagner, 1995; Threadgill et al., 1995; Chen et al., 2000).

EGF family and EGFR have also been studied in some fish species. In the goldfish ovary, EGF and TGFα showed interactive effects with gonadotropins (hCG) and insulin-like growth factors (IGFs) in stimulating DNA synthesis and EGF mediated the actions of hCG (Kumar Srivastava and Van Der Kraak, 1995). In the ovary of rainbow trout, EGF suppressed apoptosis in pre-ovulatory follicles *in vitro* (Janz and Van Der Kraak, 1997). Similar to that in mammals, EGF and TGFα also promoted oocyte maturation in fish including goldfish (Pati et al., 1996) and zebrafish (Pang and Ge, 2002). Interestingly, our previous study showed that the stimulatory effects of EGF and TGFα on zebrafish oocyte maturation could be blocked by follistatin, an activin-binding protein, suggesting a role for activin–inhibin system in mediating EGF/TGFα signaling (Pang and Ge, 2002). This was supported by the evidence that EGF stimulated expression of all three activin/inhibin β subunits (*inhbaa*, *inhbab*, and *inhbb*) in cultured ovarian follicle cells *via* EGFR but different downstream pathways (MAPK3/1 for *inhbaa/inhbb* and PI3K/Akt for *inhbab*) (Chung and Ge, 2012). In addition, we also demonstrated that EGF family members including EGF, TGFα, HB-EGF and BTC all suppressed basal and estrogen-stimulated expression of LH receptor (*lhcgr*) in cultured zebrafish follicle cells (Liu and Ge, 2013). Despite these studies on EGF activities in fish ovaries, molecular characterization of EGF family and EGFR had been limited until we cloned Egf/*egf* and Egfr/*egfr* in zebrafish, which represented the first EGF and EGFR identified and characterized in non-mammalian vertebrates (Wang and Ge, 2004a). Semiquantitative RT-PCR assays showed that EGF family members (EGF/*egf*, TGFα/*tgfa*, HB-EGF/*hbegf*, and BTC/*btc*) were primarily expressed in the oocyte with limited expression in somatic follicle cells. On the other hand, the expression of *egfr* and EGFR-induced MAPK phosphorylation were exclusively detected in the somatic follicle cells (Wang and Ge, 2004a; Tse and Ge, 2010; Chung and Ge, 2012). This distinct distribution pattern strongly suggests a potential paracrine pathway in the follicle that mediates an oocyte-to-follicle cell communication. However, the importance of this EGF ligand-EGFR communication pathway in the follicle remains entirely unknown.

Using CRISPR/Cas9-mediated gene knockout approach, we investigated the functional importance of Egf/*egf* and Egfr (*egfra* and *egfrb*) in the zebrafish with particular emphasis on their roles in reproductive performance, especially gonadal development and function. Our data demonstrated that EGF was dispensable for zebrafish reproduction despite its influence on female fecundity at older age. In contrast, the loss of EGFR (Egfra but not the truncated Egfrb) caused a complete arrest of follicle development at early stage, resulting in female infertility. We also provided evidence for a potential interaction between EGFR signaling and activin-inhibin pathway.

## Materials and methods

### Animals

The AB strain zebrafish and inhibin null mutant (*inha*−/−) used in this study were maintained in the ZebTEC Multilinking Rack zebrafish system (Tecniplast, Buguggiate, Italy) at 28°C with a lighting scheme of 14-h (8:00 am–10:00 pm) light and 10-h dark. The *inha* mutant (umo19 with ZFIN) was recently created in our laboratory (Lu et al., 2020). The fish were handled according to the guidelines and protocols approved by the Research Ethics Panel of the University of Macau.

### Establishment of zebrafish mutant lines

The *egf* and *egfra* mutants were generated by CRISPR/Cas9 gene editing method as described in our previous report (Lau et al., 2016). Briefly, the target sites were designed using the ZiFiT Targeter (version 4.2, http://zifit.partners.org/ZiFiT/Disclaimer.aspx), which identified target sequences in exon 4 and exon 6 of *egf*, and exon 7 of *egfra* genes, respectively. A target site in *egfrb* gene, a truncated form of EGFR-like molecule, was also designed based on an EST sequence as mRNA information was not found in the GeneBank. The target oligonucleotides were synthesized and cloned into the sgRNA expression vector pDR274 (Addgene #42250). The DraI-digested pDR274 sgRNA constructs were used as template to transcribe sgRNA by MAXIscript T7 kit (Thermo Fisher Scientific, Waltham, MA, USA). The Cas9 mRNA was transcribed from pCS2-nCas9n plasmid (Addgene #47929, Watertown, MA, USA) by mMESSAGE mMACHINE SP6 kit (Thermo Fisher Scientific). A mixture of 100 pg of sgRNA and 400 pg of Cas9 mRNA were co-injected into the one-cell stage embryos. All embryos were maintained in 28°C fish water. Genotyping was performed on DNA extracted from each zebrafish embryo or tail fin cut (Zhang et al., 2015b).

### High-resolution melt analysis (HRMA)

Genomic DNA was extracted by incubating the embryo or tail fin cut in 50 mM NaOH at 95°C for 12 min. After cooling to room temperature, one-tenth volume of 1 M Tris (pH 8.0) was added to neutralize the solution. Then the solution was centrifuged at 1,500 rpm for 5 min. DNA in the supernatant was used as the template for PCR. The primers for PCR were listed in Supplementary Table S1. Real-time qPCR was performed using SsoFast EvaGreen Supermix on C1000 Thermal Cycler CFX96 Real-time PCR Detection System (Bio-Rad, Hercules, CA, USA). HRMA was performed at the end of reaction with Precision Melt Analysis software (Bio-Rad) to analyze the difference of the melt curves.

### Heteroduplex mobility assay (HMA)

The PCR products from HRMA were used for HMA. They were separated on 20% nondenaturing polyacrylamide gel (20% acrylamide, 1 × TBE, 0.01% ammonium persulfate, 0.04% TEMED) at a constant voltage of 150 V for 4 h. The gel was stained with GelRed for 10 min and imaged on the ChemiDoc MP Imaging System (Bio-Rad). The mobility of heteroduplexes was slower than homoduplexes. To distinguish homozygous mutant (−/−) from wildtype (WT, +/+) in F2 generation, WT genomic DNA was added to each sample. The homozygous mutant (−/−) produces hybrid (+/−) after spiking with WT DNA, while WT (+/+) remains the same (+/+).

### Mutant selection and DNA sequencing

The F0 fish carrying mutations was crossed with WT fish to obtain the F1 generation (+/−). Different mutation patterns were identified by HRMA and HMA. The PCR products containing target sites from each pattern were cloned into pMD18-T vector (TaKaRa, Shiga, Japan) and transformed into DH5α competent *E. coli* cells. The plasmids in monoclonal bacteria were purified for DNA sequencing. The male and female fish with the same frame shift mutations were crossed to produce homozygous F2 mutants (−/−).

### Growth rate assessment

Growth rate was compared between homozygous mutant fish and their heterozygous siblings. The body weight (BW) and standard body length (BL) of males were measured every 10 days from 50 to 120 dpf, the period when zebrafish displays the highest growth rate. We chose males for growth analysis because their somatic growth was less influenced by gonadal size (*n* = 30). All data were expressed as mean ± SEM. Statistical significance was determined by one-way analysis of variance (ANOVA) followed by Tukey’s comparison test (*p* < 0.05).

### Fertility assay

The fertility of different genotypes was assessed by natural mating with WT partners. Individuals that failed to spawn after at least 10 trials were considered infertile. Once fertilized embryos were obtained, genotyping was performed on sampled embryos by HMA to confirm the genotypes of the parents.

### Fecundity assay

Fecundity of *egf* mutant females was assessed at 4 and 7 months post-fertilization (mpf). All females were separated from males for 1 week before fecundity test. Then, each *egf* ^−/−^ female fish was mated with two WT males in the spawning box (Tecniplast). Five females were examined in each test. Total egg number was counted including both fertilized and unfertilized ones. The fertilization rate was the ratio of fertilized eggs in the total. The fecundity tests were repeated five times at 4-day interval. Age-matched WT females were used as the control. The data were expressed as mean ± SEM. Statistical significance was determined using independent sample *t*-test (*p* < 0.05).

### Histological examination

All fish used for histological examination were genotyped first on DNA from the tail fin cut. Sibling WT (+/+) and/or heterozygous (+/−) fish were used as the controls. The fish was anesthetized with MS-222 (tricaine methanesulphonate, 250 mg/L; Sigma-Aldrich, St. Louis, MO, United States) and BW and BL were recorded. The body and gonad were photographed before fixing with Bouin’s solution. The samples were fixed for at least 24 h, washed with 50% ethanol, dehydrated, and embedded in paraffin. The samples were sectioned at 5 µm and stained with hematoxylin and eosin (HE) for microscopic examination.

### Gonadal-somatic index (GSI)

The body weight (W_b_) and gonad weight (W_g_) of each fish were measured at 4 mpf for homozygous mutant and their heterozygous siblings. The GSI was calculated as follows: GSI (%) = 100 * W_g_/W_b_. More than 10 fish were examined for each genotype (*n* = 11–19). Statistical significance was determined using independent sample *t*-test (*p* < 0.01).

### RNA extraction, RT-PCR, and transcriptome analysis

Tissue samples or different stage follicles (primary growth, PG; previtellogenic, PV; early vitellogenic, EV; mid-vitellogenic, MV; full-grown, FG) were collected and homogenized in 500 μl Trizol Reagent (Thermo Fisher Scientific) according to our previous reports (Wang and Ge, 2004b; Zhou et al., 2011). Total RNA was extracted from each sample according to the protocol of the manufacturer and treated with DNase I (Invitrogen, Carlsbad, CA, United States) to ensure no genomic DNA contamination. Reverse transcription was carried out at 37°C for 60 min in 10 µl reaction buffer containing 1 μg total RNA, 5 μM oligo dT primer, 0.75 mM deoxynucleotide triphosphate mixture, and 200 U M-MLV reverse transcriptase (Thermo Fisher Scientific). The reaction was inactivated by heating at 70°C for 15 min. Quantitative PCR (qPCR) reactions were performed on the CFX96 Real-Time PCR Systems using SsoFast EvaGreen Supermix (Bio-Rad). The expression levels of target genes during folliculogenesis were first normalized to that of the housekeeping gene *ef1a* and then expressed as the fold change relative to that at the PG stage. The primers used for cDNA amplification were designed using Primer Premier 6 (Supplementary Table S1). Each experiment was performed at least twice in triplicate and all values were expressed as mean ± SEM. The qPCR data were analyzed by ANOVA followed by Tukey’s comparison test (*p* < 0.05).

To compare ovarian transcriptomes between *egfra* ^+/−^ and *egfra* ^−/−^, the ovaries of 45-dpf females were selected for RNA-seq analysis. Three fish with BL at 1.8 cm and BW at 100 mg were sampled for each genotype (*n* =3 biological replicates). The fish were anesthetized and the whole ovary from each fish was homogenized for RNA extraction. RNA-seq and data analysis were performed by Novogene Bioinformatics Technology (Tianjin, China). The sequencing of each sample generated approximately 4G raw data, which have been submitted to the NCBI SRA database with accession numbers SRR12432918–12432923. TopHat2 algorithm was chosen to map reads to the zebrafish genome, and FPKM (Fragments Per Kilobase Million) was used to normalize the expression data followed by expression level estimation and differential expression analysis by HTSeq and DESeq software (padj <0.05). Gene ontology (GO) analysis was performed on genes that showed twice higher or lower expression in the ovary of *egfra−/−*fish than that of *egfra+*/*−*fish.

### Epidermal growth factor treatment and Western blotting

The ovaries of 45-dpf *egfra* ^+/−^ and *egfra* ^−/−^ females (*n* = 3; ∼1.8 cm BL and ∼100 mg BW) were dissected out, dispersed and incubated in M199 medium for 30 min. The medium was then replaced with fresh M199 containing 200 nM recombinant human EGF (PeproTech, Rochy Hill, NJ, USA). The medium was removed after 20 min treatment. The treated ovarian fragments and follicles were homogenized with a pestle in cold SDS sample buffer (63 mM Tris-HCl pH 6.8, 10% glycerol, 5% β-mercaptoethanol, 3.5% sodium dodecyl sulfate, 1% w/v SDS). The samples were centrifuged on a microfuge at the highest speed for 5 min. The supernatant was collected and mixed with loading buffer followed by heating at 95°C for 10 min. The samples were separated on 12% polyacrylamide gels and transferred to PVDF membranes. The membranes were blocked with 5% nonfat dry milk (Bio-Rad) in 1× TBST at room temperature for 1 h. After rinsing once with 1× TBST, the membranes were incubated at 4°C overnight in 1× TBST with 2% BSA and primary antibodies for p-Erk1/2 (1:2,000; #4370) and Erk1/2 (1:1,000; #9102) (Cell Signaling Technology, Danvers, MA, United States). The membranes were then washed with 1× TBST three times followed by incubation with HRP-conjugated anti-rabbit IgG (1:5,000; #7074) (Cell Signaling) at room temperature for 1 h. After washing, the membranes were incubated with ECL Western Blotting Substrate (Thermo Scientific Pierce), and images were detected on the ChemiDoc MP Imaging System (Bio-Rad).

### Immunohistochemistry

The paraffin sections were deparaffinized in xylene and rehydrated in gradient ethanol and water. Antigen retrieval was performed in 10 mM sodium citrate buffer at sub-boiling temperature for 10 min. The endogenous hydrogen peroxidase was inactivated by treatment with 3% hydrogen peroxide (H_2_O_2_) for 10 min. The sections were washed with 1× PBS three times for 5 min each before blocking for 1 h at room temperature with normal horse serum. Each slide was then incubated at 4°C overnight with 100 μl of p-MAPK3/1 antibody (#4370, Cell Signaling) diluted at 1:100 in blocking solution. The section was then washed with 1× PBS three times for 5 min each before incubation with 100 μl of HRP-linked anti-rabbit IgG antibody (#7074, Cell Signaling) for 30 min at room temperature. After washing with 1× PBS three times for 5 min each, 100 μl of DAB solution was added to each section and incubated for 10 min. The section was washed with tap water for 5 min to stop reaction, dehydrated, and mounted with Permount (Thermo Fisher Scientific).

### Data analysis

The mRNA level of target gene was determined by qPCR, normalized to the housekeeping gene *ef1a*, and expressed as fold change relative to the control group. The expression level of each gene in transcriptome analysis was expressed as reads per kilobase of exon model per million mapped reads (FPKM). All values were expressed as mean ± SEM. Statistical analysis was carried out with Prism 5 (GraphPad, San Diego, CA, USA), and one-way ANOVA was used to analyze gene expression or fish growth rate. The GSI and fertilization rate were analyzed by *t*-test. All experiments were performed at least twice.

## Results

### Spatiotemporal expression of *egf*, *egfra*, and *egfrb* in gonads and non-gonadal tissues

In addition to EGFR (Egfra*/egfra*) that we characterized previously in zebrafish (Wang and Ge, 2004a), another putative form of EGFR (Egfrb*/egfrb*) is also present but not well annotated in the zebrafish genome. Homology and phylogenetic analysis showed that *egfra* and *egfrb* are related and positioned in the same cluster. Egfra is more closely related to EGFR of other fish whereas Egfrb is remotely positioned in the cluster (Supplementary Figure S1A). Protein sequence alignment showed that Egfrb shared high homology with the extracellular domain of Egfra, but it ended at or before the transmembrane domain, representing a truncated form (Supplementary Figure S1B).

**FIGURE 1:**
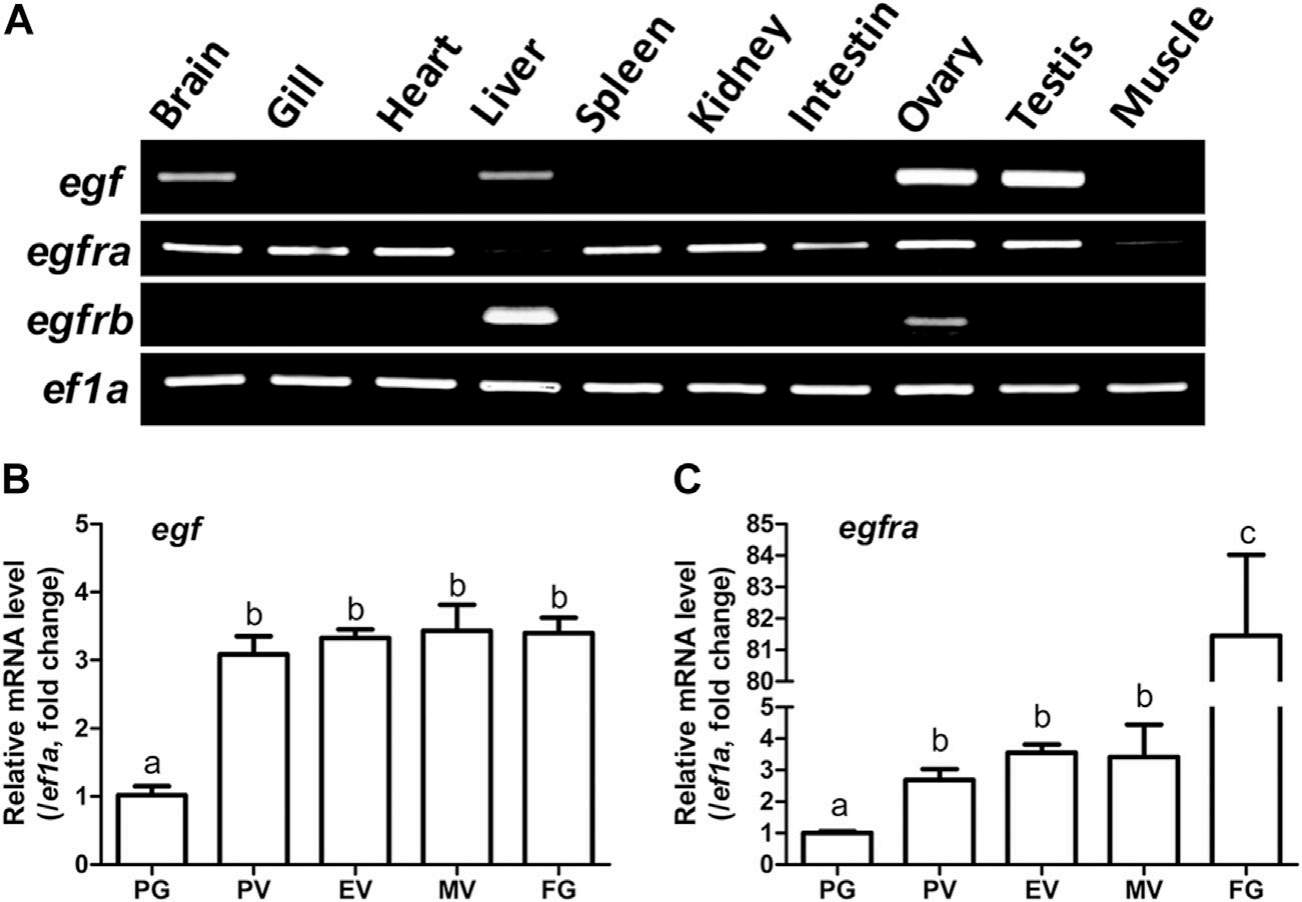
Tissue distribution of *egf*, *egfra,* and *egfrb,* and temporal expression profiles of *egf* and *egfra* during folliculogenesis. **(A)** RT-PCR analysis showed that *egf* was mainly expressed in the ovary and testis with weak expression in the brain, liver, and gill, whereas *egfra* was expressed in all organs, but with low levels in the liver and muscle. In contrast, *egfrb* was exclusively expressed in the liver and ovary with higher level in the liver. **(B,C)** The expression of *egf* and *egfra* mRNA was low in the PG follicles and increased significantly in the PV follicles. The mRNA level of *egf* maintained relatively constant after PV stage, whereas the expression of *egfra* showed a second surge in FG follicles prior to maturation. The expression levels of target genes were normalized to that of housekeeping gene *ef1a*, and expressed as fold change compared with that in PG follicles. Different letters indicate statistical significance (*n* = 3). PG, primary growth; PV, previtellogenic; EV, early vitellogenic; MV, mid-vitellogenic; FG, full-grown. EGF/*egf*, epidermal growth factor; EGFR/*egfr*, epidermal growth factor receptor.

RT-PCR analysis showed that *egf* was predominantly expressed in the ovary and testis with weak expression in some other tissues such as the brain and liver. However, *egfra* exhibited ubiquitous expression in various tissues investigated with low expression in the liver and muscle. These agree well with our previous reports (Wang and Ge, 2004a; Tse and Ge, 2010). Interestingly, *egfrb* also showed distinct tissue distribution with expression detectable only in liver and ovary, but not in other tissues (Figure 1A).

We then analyzed the temporal expression profiles of *egf* and *egfra* during folliculogenesis in sexually mature females. Quantitative PCR analysis demonstrated that *egf* and *egfra* mRNA levels were relatively low in primary growth (PG, stage I) follicles. When PG follicles were activated or recruited into pre-vitellogenic (PV, stage II) stage, their expression levels were both significantly increased and remained high during vitellogenic growth from PV to full-grown (FG) stage (stage III) (Figures 1B, C). Interestingly, *egfra* but not *egf* showed a further dramatic increase in expression in FG follicles prior to final oocyte maturation (Figure 1C). These results were consistent with our previous report using real-time RT-PCR (Tse and Ge, 2010).

### Establishment of *egf*, *egfra*, and *egfrb* mutants in zebrafish

We generated loss-of-function mutants by CRISPR/Cas9 method, which targeted the coding region downstream of the translation start codon to knock out the protein by frameshifting indel mutations. To disrupt *egf*, the target site was located in exon 4 of the gene and a mutant containing a 2-bp deletion was selected for phenotype analysis (*egf*−/−; ZFIN line number: umo21). This frameshifting mutation introduced a stop codon near the target site that resulted in translation of a truncated fragment. To verify the loss of *egf* gene, we analyzed the expression of *egf* transcript in the ovary by using a mutant-specific primer (F2) with its 3′-end located in the deletion region. As shown in Supplementary Figure S2A, the primer pair (F1/R1) that flanks the deletion site could amplify signals in both WT (*egf*+/+) and mutant fish (*egf*−/−); however, the primer pair F2/R1 could only detect the signal in WT fish (Supplementary Figure S2A). For *egfra* gene, the target site was located in exon 7 and a 4-bp deletion mutant was identified (*egfra*−/−; umo22). The 4-bp deletion caused frameshift and translation of a truncated product. We used similar RT-PCR approach to demonstrate the mutation at the transcript level (Supplementary Figure S2B). We also generated a mutant line for *egfrb* gene with 11-bp deletion (umo23) (Supplementary Figure S2C).

### Growth performance of *egf* and *egfra* mutants

**FIGURE 2:**
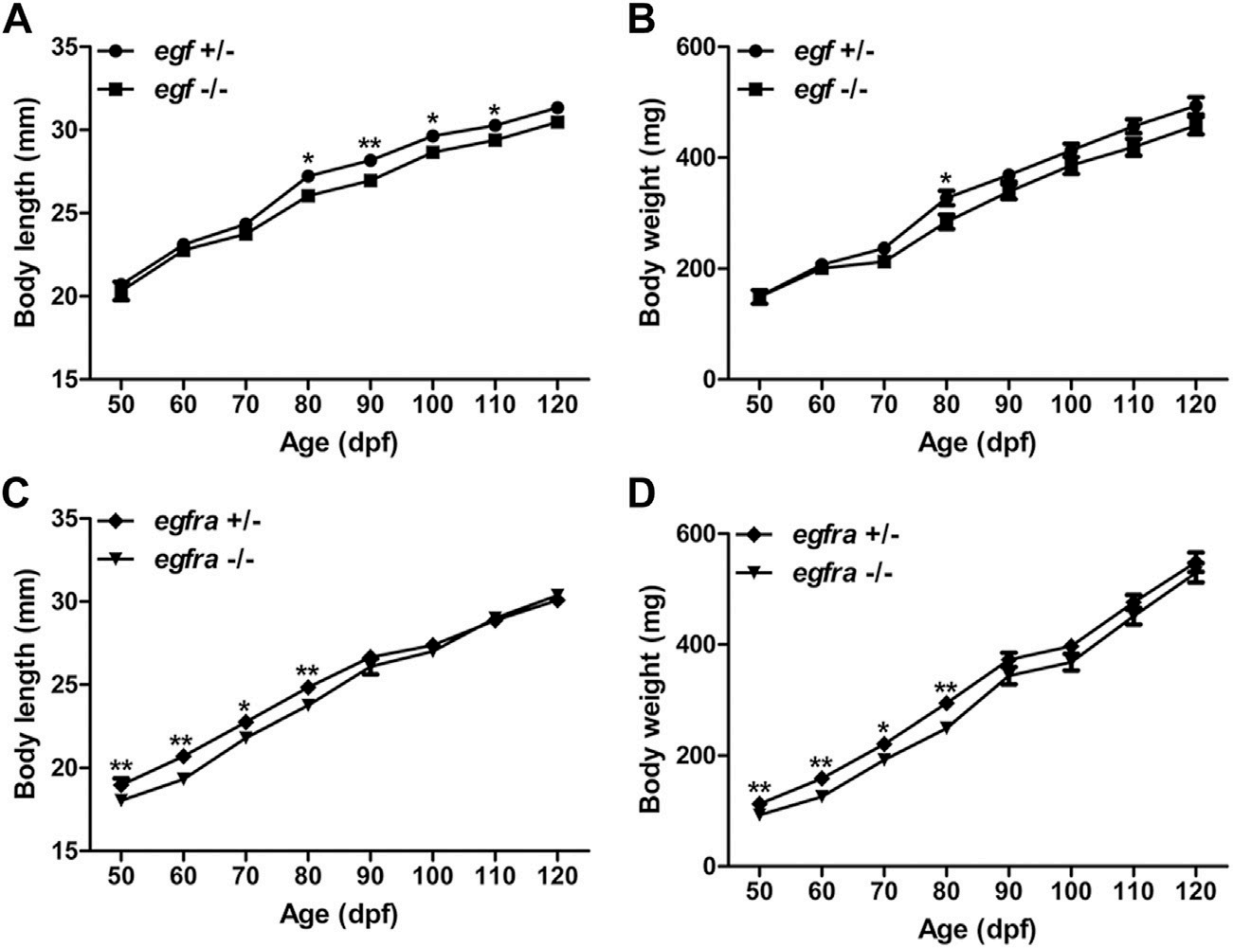
Growth performance of *egf* and *egfra* mutant males. **(A,B)** The standard body length and body weight of *egf*−/− fish were comparable with those of *egf*+/− fish with slight decrease after 70 dpf. **(C,D)** The standard body length and body weight of *egfra*−/− fish were slightly below those of *egfra*+/− fish from 50 to 80 dpf, and they became comparable after 90 dpf. *p < 0.05; **p < 0.01 (*n* = 19–50).

Considering that EGF is a well-known growth factor involved in cell growth and proliferation, we first compared the growth rate of the homozygous mutant males (*egf*−/−) with their heterozygous male siblings (*egf*+/−) from 50 to 120 days post-fertilization (dpf). We chose males for growth analysis because their somatic growth was less influenced by gonadal development. The BW and BL of the mutant fish (*egf−/−*) were comparable with those of their heterozygous siblings (*egf*+/−) from 50 to 70 dpf. However, the growth of *egf*−/− mutant slowed down slightly after 70 dpf with BL being slightly but significantly shorter than that of *egf*+/− fish from 80 to 110 dpf (Figure 2A) and BW lower than that of the heterozygous siblings from 70 to 120 dpf although statistical significance was only detected at 80 dpf (Figure 2B). Similarly, both BL and BW of the homozygous *egfra*−/− mutant were slightly but significantly lower than those of heterozygous fish (*egfra*+/−) from 50 to 80 dpf; however, the difference diminished afterward (Figures 2C, D).

### Effects of *egf* mutation on fertility and gametogenesis

**FIGURE 3:**
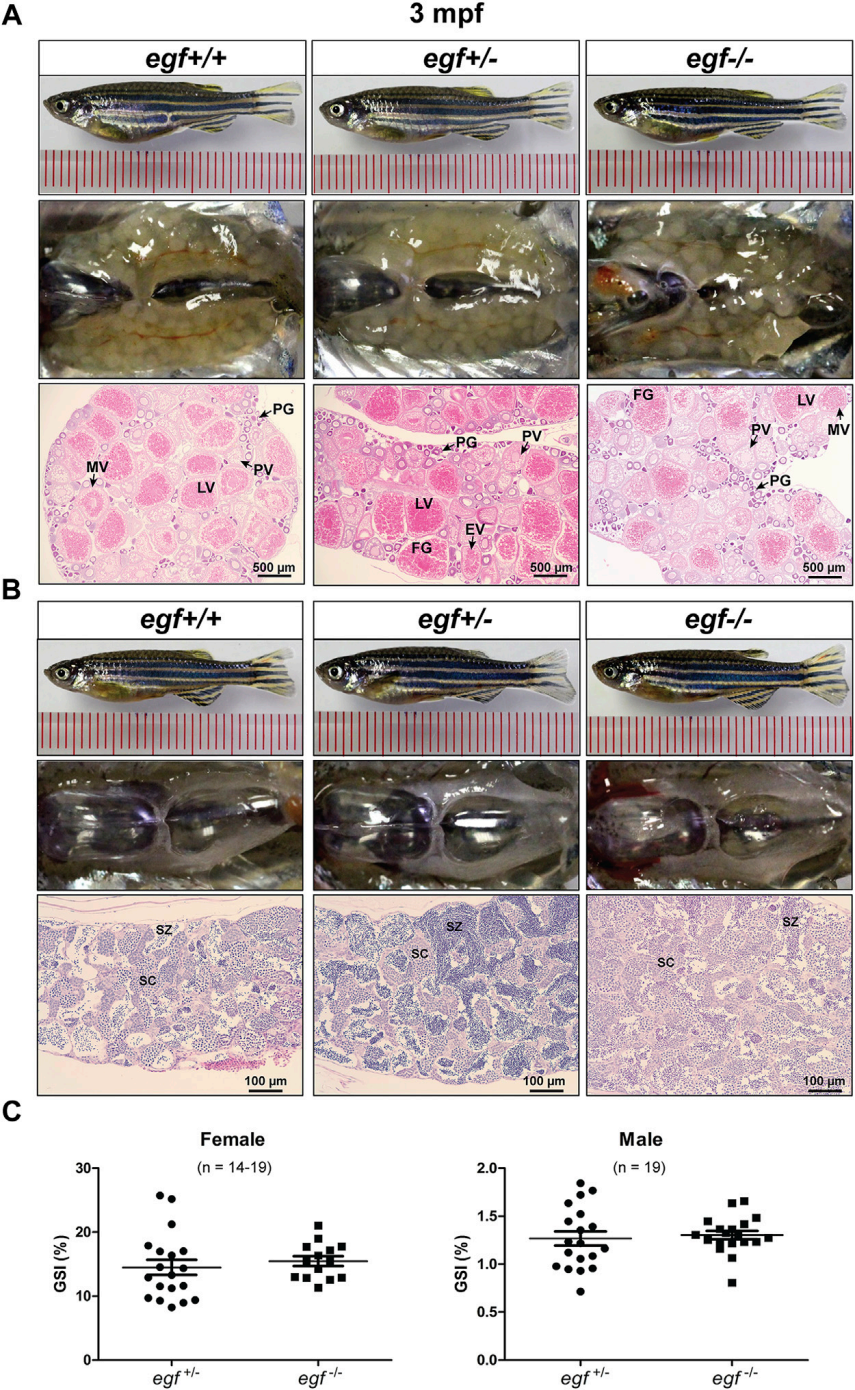
Gonadal development of *egf* mutant at 3 mpf. **(A)** Anatomical and histological examination of the ovary in *egf* mutant (*egf*−/−) and controls (*egf*+/+ and *egf*+/−). The *egf*-deficient follicles developed normally. **(B)** Anatomical and histological examination of the testis in *egf* mutant (*egf*−/−) and controls (*egf*+/+ and *egf*+/−). The spermiogenesis was normal in *egf*-deficient males. **(C)** GSI in females (*n* = 14–19) and males (*n* = 19). PG, primary growth; PV, previtellogenic; EV, early vitellogenic; MV, mid-vitellogenic; LV, late vitellogenic; FG, full-grown; SC, spermatocytes; SZ, spermatozoa.

Our previous studies showed that EGF was most abundantly expressed in zebrafish gonads (ovary and testis) (Wang and Ge, 2004a; Tse and Ge, 2010) and that it stimulated oocyte maturation *in vitro* (Pang and Ge, 2002). The expression pattern was confirmed in this study (Figure 1A). After demonstrating that the loss of *egf* only had slight effect on somatic growth, we turned our attention to its potential impact on gonadal development and function. The mutant *egf*−/− fish had normal fertility (fecundity and fertilization rate) at 3 mpf in both females and males compared with that of control fish (*egf*+/−) (data not shown). Histological examination demonstrated well-developed follicles of all stages in the ovaries of both mutant (*egf*−/−) and control (*egf*+/+ and *egf*+/−) females (Figure 3A) and normal spermatogenesis in the testes of all three genotypes as well (Figure 3B). In agreement with this, GSI was also comparable between *egf*+/− and *egf*−/− fish in both sexes (Figure 3C). To confirm these results, we also examined another *egf* mutant line with 44-bp deletion (Supplementary Figure S3; umo24); it also showed normal folliculogenesis and spermatogenesis (Supplementary Figure S4).

**FIGURE 4:**
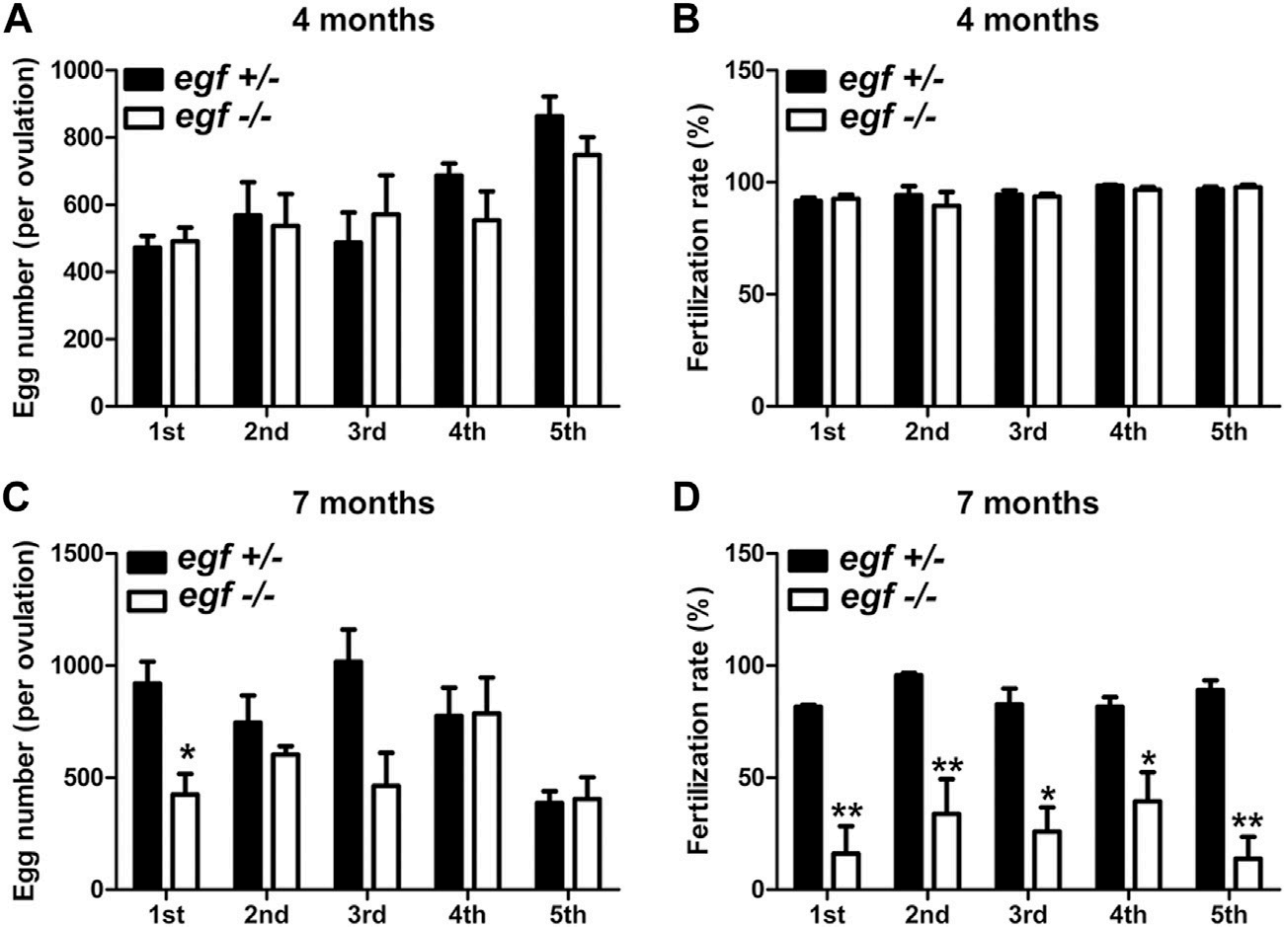
Reproductive performance of *egf* mutant at 4 and 7 mpf. **(A)** Egg numbers at 4 mpf in five fecundity tests (1st–5th). **(B)** Fertilization rates in five spawning tests at 4 mpf. **(C)** Egg number per spawning at 7 mpf. **(D)** Fertilization rate at 7 mpf. *p < 0.05; **p < 0.01 (*n* = 5 per test).

The fecundity of *egf*-deficient females was tested by crossing with the WT males. As shown in Figure 4, the number of eggs produced in each spawning (fecundity) and the fertilization rate were comparable between the control (*egf*+/−) and mutant (*egf*−/−) at 4 mpf (Figures 4A, B). However, the mutant females produced less eggs at 7 mpf in most trials and the fertilization rates decreased significantly in all tests (average 25% vs. 87%) (Figures 4C, D).

### Impact of epidermal growth factor receptor signaling on gonadal development and gametogenesis

The lack of substantial impact of *egf* mutation on development and reproduction was surprising given that *egf* is most abundantly expressed in the gonads. Considering that multiple EGF family ligands can bind to the same EGF receptor (EGFR, ERBB-1, or HER1 in humans), we hypothesized that the lost EGF function in the *egf* mutant could have been compensated by other family members. To address this issue, we went on to delete EGF receptor gene (Egfr/*egfr*) to disrupt the signaling pathway. There were two paralogs of *egfr* gene in zebrafish genome (*egfra* and *egfrb*), and *egfrb* is a truncated form that has not been characterized. A mutant line of *egfra* with 4-bp deletion and a line with 11-bp deletion for *egfrb* were chosen for phenotype analysis.

**FIGURE 5:**
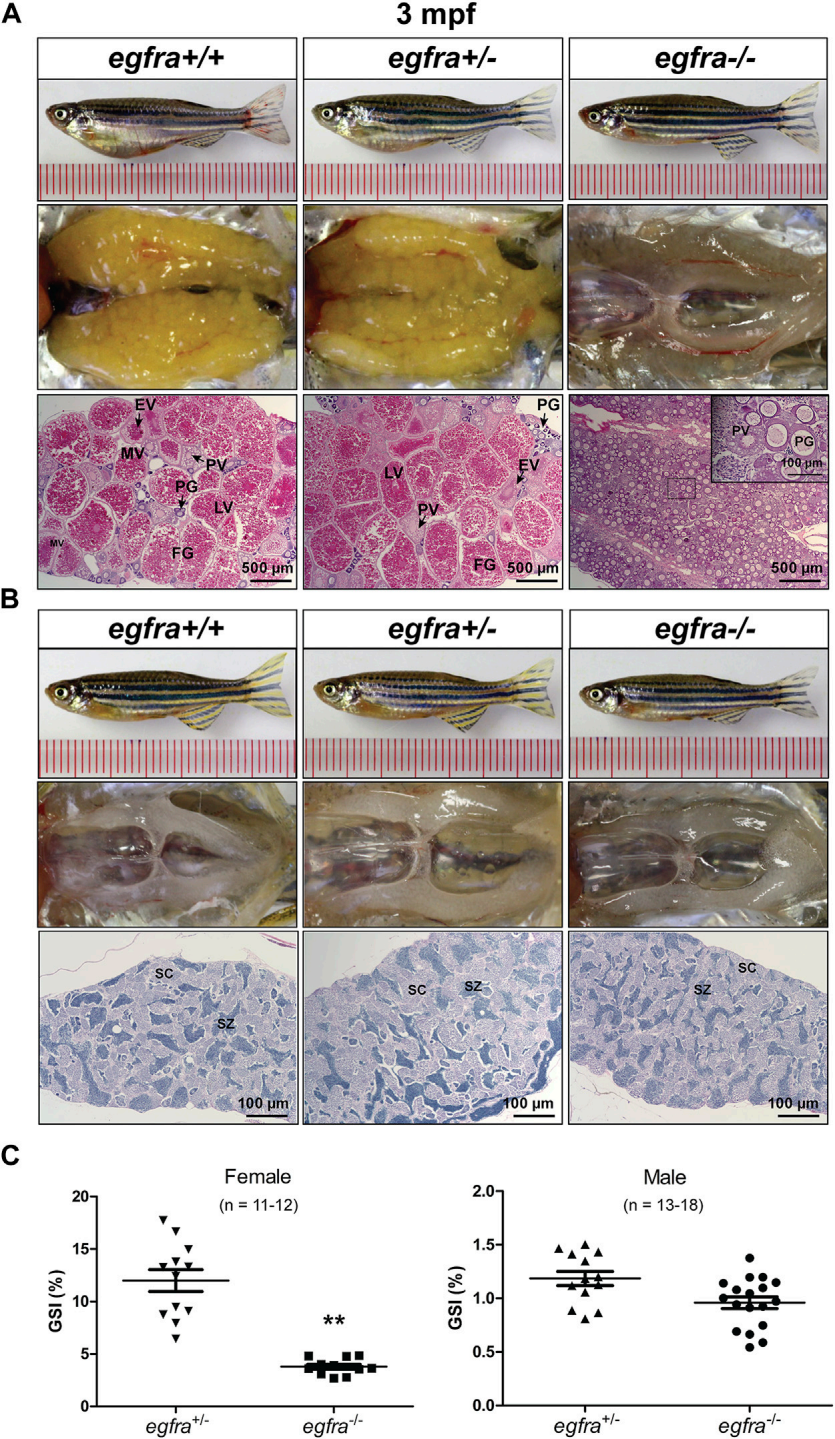
Gonadal development of *egfra* mutant at 3 mpf. **(A)** Anatomical and histological examination of the ovary in *egfra* mutant (*egfra*−/−) and controls (*egfra*+/+ and *egfra*+/−). The follicles of *egfra*-deficient females were arrested at PG stage with only a few entering early PV stage. **(B)** Anatomical and histological examination of the testis in *egfra* mutant (*egfra*−/−) and controls (*egfra*+/+ and *egfra*+/−). The spermiogenesis was normal in *egfra* mutant males. **(C)** GSI of *egfra*-deficient females (*n* = 11–12) and males (*n* = 13–18). The GSI of female mutant was much lower than that of control whereas no difference was found in males (**p < 0.01). PG, primary growth; PV, previtellogenic; EV, early vitellogenic; MV, mid-vitellogenic; LV, late vitellogenic; FG, full-grown; SC, spermatocytes; SZ, spermatozoa.

The female mutant (*egfra*−/−) exhibited feminine secondary sexual characteristics at 3 mpf, such as silverish body color; however, it showed slim body shape compared with the controls (*egfra*+/+ and (*egfra*+/−). When crossing with WT males, all *egfra*−/− females tested failed to spawn. Dissection revealed that the mutant ovaries (*egfra*−/−) were translucent and immature, in contrast to the control ovaries (*egfra*+/+ and *egfra*+/−), which were full of normal vitellogenic follicles including FG stage. Histological examination showed that the *egfra*−/− follicles were primarily arrested at the PG stage with only a few entering very early PV stage with some small cortical alveoli in the cytoplasm (Figure 5A).

By comparison, no obvious defects in fertility were observed in *egfra*−/− mutant males, which could produce viable offspring when crossing with WT (*egfra*+/+) and heterozygous (*egfra*+/−) females. Histological examination showed that the testes were structurally normal in *egfra*−/− males compared with *egfra*+/+ and *egfra*+/− males. The testicular lumens were well developed with similar amounts of spermatozoa in *egfra*−/− (Figure 5B). Measurement of GSI showed that the ovaries of *egfra−/−* mutant females were significantly smaller than those of *egfra*+/- females; however, it was comparable between mutant (*egfra−/−*) and control (*egfra*+/−) males (Figure 5C).

Similar to *egfra*, *egfrb* was also expressed in the ovary (Figure 1). In contrast to *egfra*, mutation of *egfrb* (−11-bp) did not cause any abnormal phenotypes and *egfrb* ^−/−^ mutant showed normal folliculogenesis and spermatogenesis in adults (90 dpf) (Supplementary Figure S5).

### Importance of *egfra* in follicle activation or primary-secondary growth transition

**FIGURE 6:**
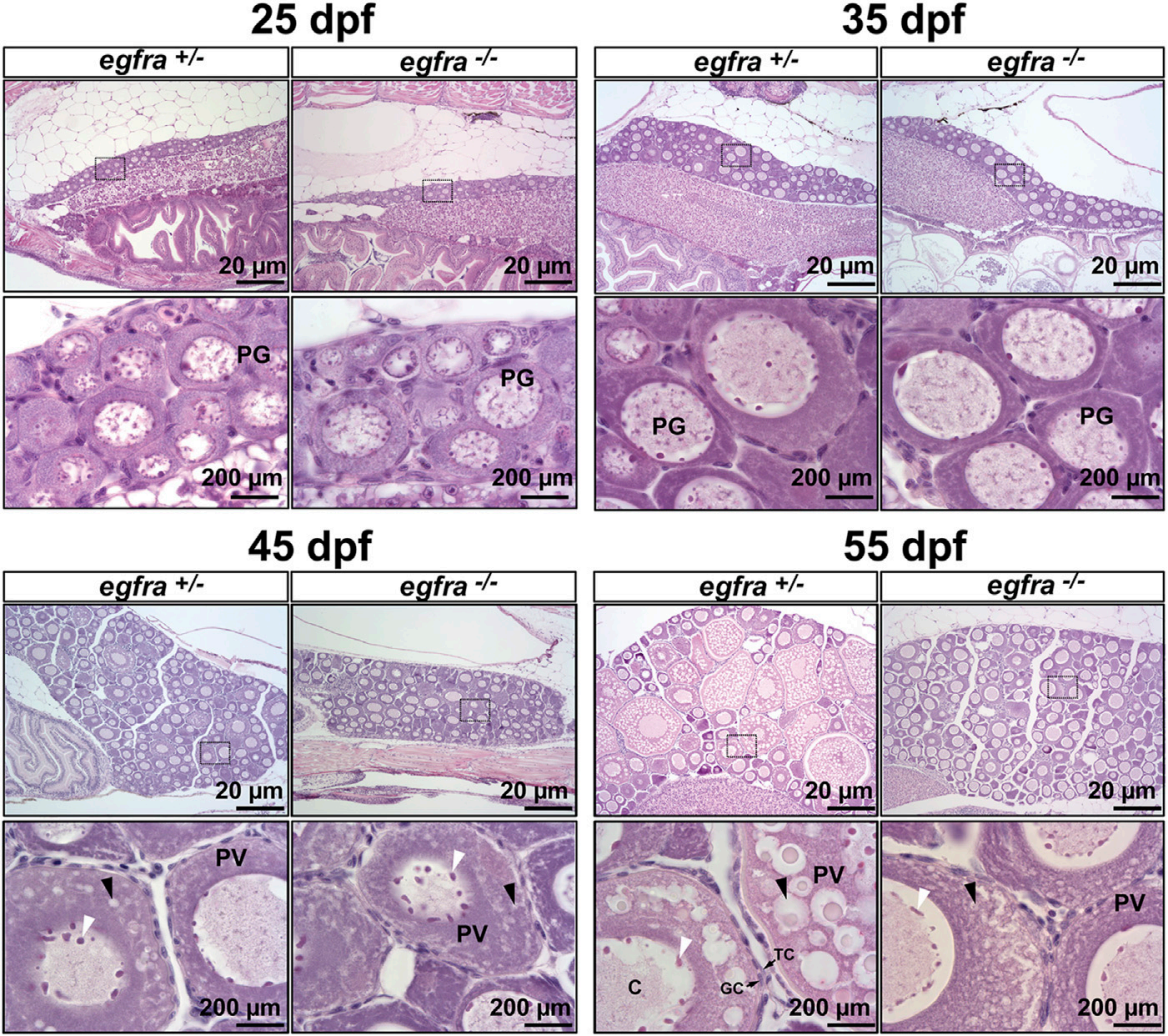
Effects of *egfra* deficiency on folliculogenesis in juvenile fish ovary. At 25 dpf, the presumptive perinucleolar oocytes at PG stage could be seen in both control (*egfra*+/−) and mutant fish (*egfra*−/−). These oocytes continued to grow in size in both genotypes at 35 dpf. At 45 dpf when puberty onset occurs in female zebrafish females, PV follicles with small cortical alveoli started to appear in the oocytes of both control (*egfra*+/−) and mutant fish (*egfra*−/−). However, the PV follicles in the control continued to grow with increasing number and size of the cortical alveoli as seen at 55 dpf, whereas the growth ceased completely in the mutant with some follicles containing rudimental cortical alveoli only. Six fish were sampled at each time point for each genotype. PG, primary growth; PV, previtellogenic; black arrow, cortical alveoli; white arrow, nucleoli at the periphery of the germinal vesicle; C, chromatin threads in the germinal vesicle; GC, granulosa cells; TC, theca cells.

To further investigate the impact of *egfra* mutation on folliculogenesis, we performed a time course examination of females at different time points of growth and development at 10-day intervals, starting from 25 dpf when zebrafish gonadal sex differentiation started (Maack and Segner, 2003; Chen and Ge, 2013; Qin et al., 2018). At 25 dpf, the ovaries in both *egfra*+/− and *egfra*−/− fish contained cystic chromatin nucleolar (CN) oocytes and emerging perinucleolar (PN) oocytes with thread-like chromatin distributed throughout the karyoplasm, representing presumptive juvenile ovary. The presence of PN follicles indicated normal cyst breakdown or follicle assembly from nested oocytes. At 35 dpf, the oocytes in both *egfra*+/− and *egfra*−/− had increased significantly in size and most of them had developed into typical PG follicles at PN stage, which is characterized by multiple nucleoli located at the periphery of the germinal vesicle and a layer of somatic cells surrounding each individual oocyte. At 45 dpf, some of the oocytes in *egfra*+/−fish significantly increased in size with one or multiple layers of small cortical alveoli appearing at the periphery of ooplasm, marking their entry into the PV stage. A thin layer of somatic follicle cells was formed immediately around oocytes. In contrast, the *egfra*−/− oocytes were mostly at the PG stage. Cortical alveoli also appeared in some mutant oocytes, but with less number and smaller size. At 55 dpf, the oocytes in control females (*egfra*+/−) underwent extensive growth with abundant and multiple layers of large cortical alveoli. In sharp contrast, the oocytes in mutant females (*egfra*−/−) remained arrested at PG stage with only a few entering early PV stage that contained small cortical alveoli (Figure 6).

### Loss of MAPK3/1 (ERK1/2) responsiveness to epidermal growth factor in *egfra*−/− ovary

**FIGURE 7:**
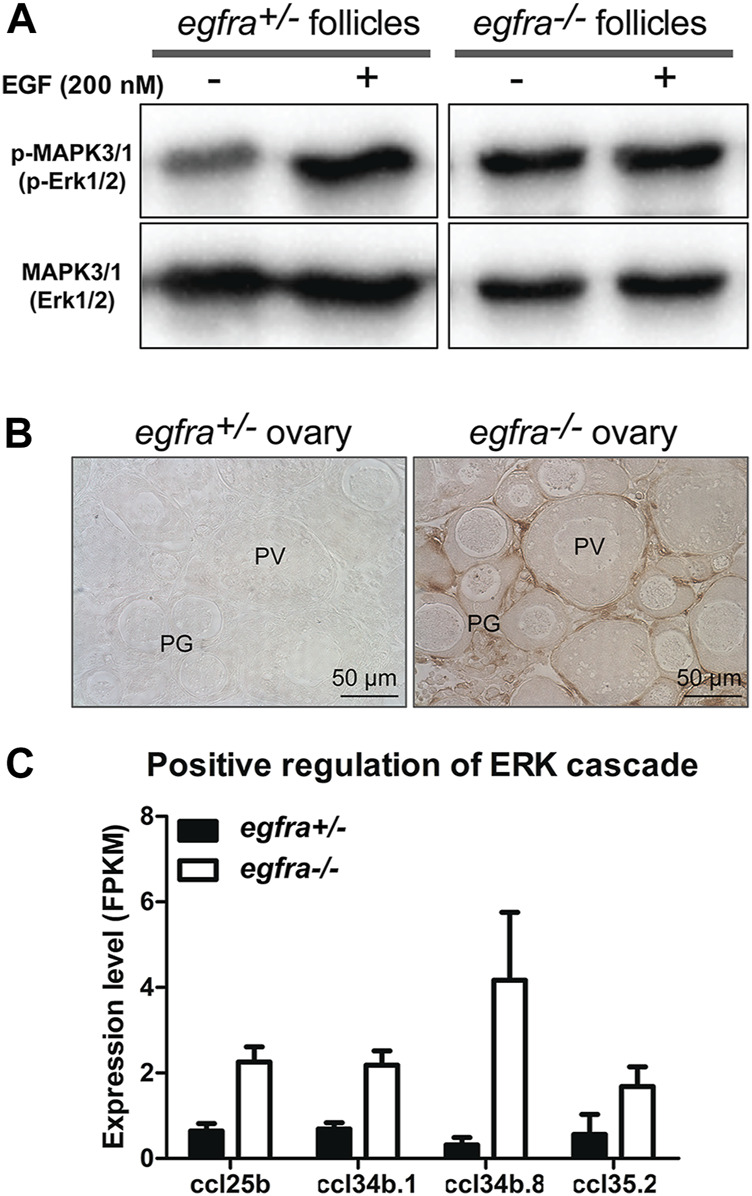
Loss of MAPK signaling response to EGF in *egfra* mutant ovary. **(A)** EGF treatment of ovarian fragments *in vitro* induced phosphorylation of MAPK3/1 (Erk1/2) in *egfra*+/− but not *egfra*−/− follicles. However, the mutant ovary showed higher basal level of MAPK phosphorylation despite its lack of response to EGF. **(B)** Immunohistochemical staining for MAPK3/1 phosphorylation in the control and mutant ovaries without EGF treatment. High level of phosphorylated MAPK3/1 was located in the somatic follicle cells of the *egfra*−/− ovary. **(C)** Increased expression of chemokine ligands (*ccl25b*, *ccl34b.1*, *ccl34b.8*, and *ccl35.2*) in *egfra*−/− follicles.

MAPK3/1 (ERK1/2) is the major pathway in zebrafish ovary that transmits EGF/EGFR signaling (Chung and Ge, 2012). To further confirm the loss of *egfra* gene in the mutant, we analyzed phosphorylation of MAPK3/1 in the follicles from both control (*egfra*+/−) and mutant (*egfra*−/−) at 45 dpf and its response to EGF treatment. The ovaries were dispersed into fragments and incubated with EGF (200 nM) for 20 min followed by Western blot analysis for MAPK3/1 phosphorylation. As expected, the control follicles (*egfra*+/−) showed a strong response to EGF with increased MAPK3/1 phosphorylation. In contrast, no response was observed in mutant follicles (*egfra*−/−). Interestingly, despite its lack of response to EGF, the mutant follicles showed higher basal levels of MAPK3/1 phosphorylation, which was similar to the EGF-induced level in the control (Figure 7A). This was further confirmed by immunohistochemical staining for phosphorylated MAPK3/1 in the ovary. Strong basal signals could be detected in sections of the mutant ovary without EGF treatment, but not the control, and the staining was mostly localized to the somatic follicle cells surrounding the oocytes (Figure 7B). Transcriptome (see below) and RT-PCR analyses demonstrated significant upregulation of chemokine ligand genes involved in positive regulation of ERK cascade, including *ccl25b*, *ccl34b.1*, *ccl34b.8*, and *ccl35.2* (Figure 7C). The increased expression of these genes might be responsible for the increased basal MAPK3/1 phosphorylation observed in *egfra* null ovary (*egfra*−/−).

### Transcriptome analysis of *egfra*+/− and *egfra*−/− follicles at follicle activation

**FIGURE 8:**
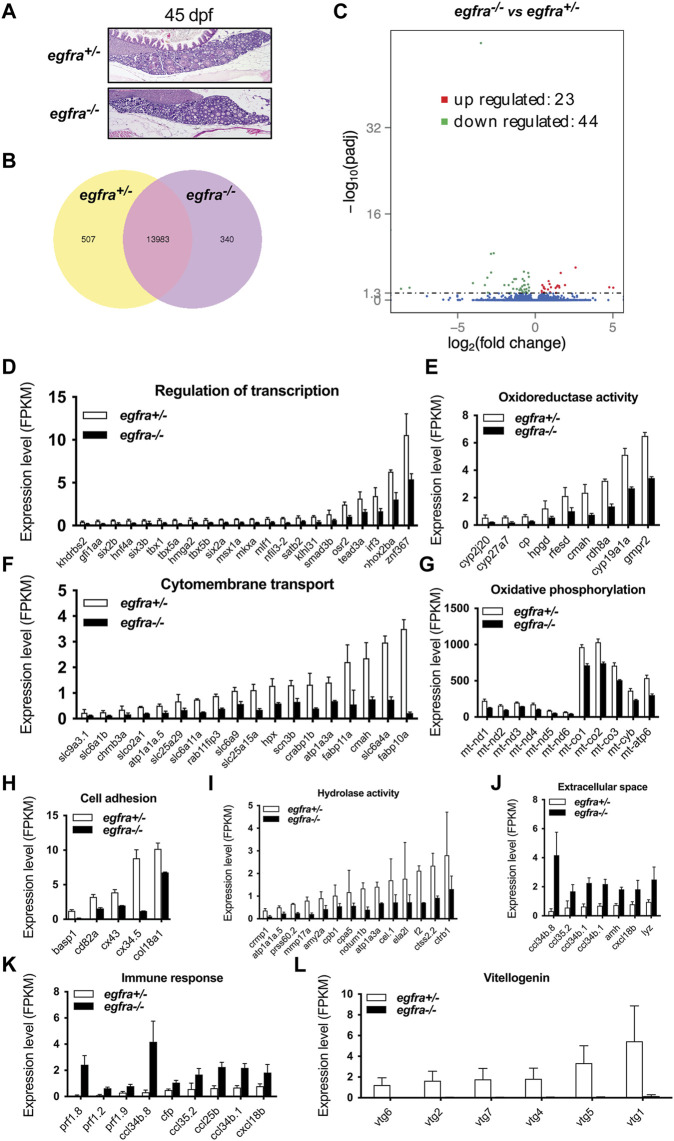
Transcriptome analysis for differentially expressed genes (DEG) in *egfra*−/− zebrafish ovary. **(A)** The ovaries of control (*egfra*+/−) and mutant (*egfra*−/−) at 45 dpf when follicle activation or PG-PV transition occurs. **(B)** Venn diagram illustrating DEGs in *egfra*+/− and *egfra*−/− ovaries. FPKM was used to normalize gene expression levels with FPKM >1 being the expression threshold. In total, 13,983 genes were expressed in the ovaries of both *egfra+/−* and *egfra−/−* fish, whereas 507 and 340 genes were only expressed in the ovaries of *egfra+/−* and *egfra−/−* fish, respectively. **(C)** MA plot showing the significantly downregulated (in green) and upregulated (in red) genes. **(D–I)** Enrichment of downregulated genes in transcription regulation, oxidoreductase activity, cytomembrane transport, oxidative phosphorylation (mitochondrial metabolic pathway), cell adhesion, and hydrolase activity. **(J,K)** Enrichment of upregulated genes in extracellular space and immune response. **(L)** Loss of expression of vitellogenin genes (*vtg1*, *vtg2*, *vtg4*, *vtg5*, *vtg6*, and *vtg7*) in mutant ovary follicles (*egfra*−/−). FPKM, fragments per kilobase million.

To understand the molecular mechanisms underlying Egfr regulation of follicle activation, we performed a transcriptome analysis on *egfra*+/− and *egfra*−/− follicles at 45 dpf when the first wave of follicle transition from PG to PV occurs. Histological examination showed that most follicles in *egfra*+/− ovaries were at PG stage with a few entering PV stage (body size: ∼1.90 cm BL; ∼138 mg BW). Similarly, most follicles in *egfra*−/− ovaries were also at PG stage with some at early PV stage (body size: ∼1.90 cm BL; ∼132 mg BW) (Figure 8A). Transcriptome analysis showed that 5.7% (847/14,830) of the genes detected were differentially expressed between *egfra*+/− and *egfra*−/− follicles (Figure 8B). Among the differentially expressed genes, 44 genes were significantly downregulated and 23 were upregulated in *egfra*−/− ovaries compared with *egfra*+/− ovaries (padj < 0.05) (Figure 8C **;** Supplementary Table S2).

GO analysis revealed that genes involved in transcription regulation, oxidoreductase activity, cytomembrane transport, oxidative phosphorylation, cell adhesion, and hydrolase activity were downregulated in *egfra*−/− ovary (Figures 8D–I **)**, whereas those in extracellular space and immune response were upregulated (Figures 8J, K).

Interestingly, several genes of great importance in gonadal development and function showed significant changes in expression. In particular, *cyp19a1a* (ovarian aromatase) (Figure 8E) and *ar* (androgen receptor) (Supplementary Table S2) were significantly downregulated, whereas *amh* (anti-Mullerian hormone) (Figure 8J and Supplementary Table S2), a male-promoting gene, was upregulated in *egfra*−/− follicles. Aromatase is responsible for producing estrogens, and androgen receptor-mediated signaling plays an important role in stimulating follicle development, including promoting granulosa cell proliferation (Laird et al., 2017; Franks and Hardy, 2018). In addition, several vitellogenin genes were found to express in control ovaries (*egfra*+/−), including *vtg1*, *vtg2*, *vtg4*, *vtg5*, *vtg6*, and *vtg7*; however, none of these genes showed expression in mutant ovaries (*egfra*−/−) (Figure 8L).

### Sexual reversal of *egfra*-deficient females to males

**FIGURE 9:**
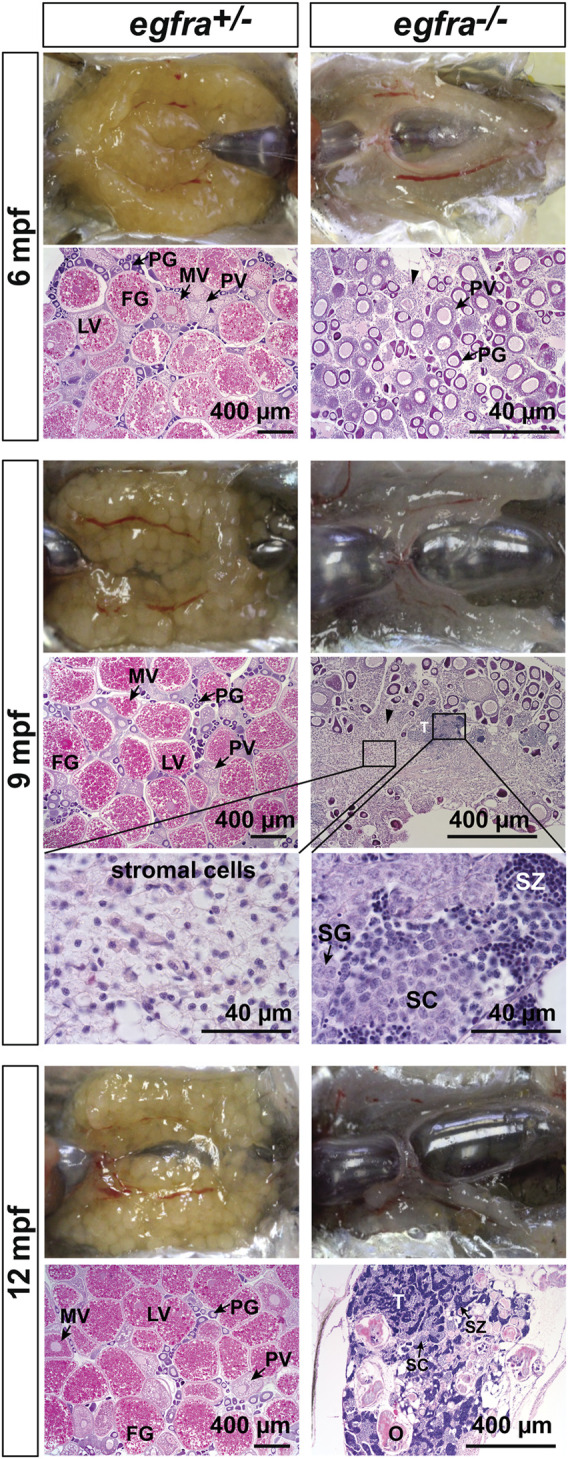
Progressive degeneration of follicles and sex reversal to males in *egfra*−/− females. Follicles in *egfra*−/− ovaries underwent progressive degeneration from 6 to 12 mpf. While the follicles were degenerating, the inter-follicular spaces were gradually occupied by somatic stromal cells, an early sign of masculinization. Testicular tissues with spermatogenic cells started to appear at 9 mpf and increased progressively afterward. By comparison, the ovaries of control *egfra*+/− females were filled with follicles of all developmental stages from PG to FG during the period. PG, primary growth; PV, previtellogenic; MV, mid-vitellogenic; LV, late vitellogenic; FG, full-grown; SG, spermatogonia; SC, spermatocytes; SZ, spermatozoa; T, testis tissue; O, degenerating oocytes.

To investigate the fate of those follicles arrested at PG stage in *egfra*−/− females, we continued to monitor the state of ovaries over 1 year at 6, 9, and 12 mpf (Figure 9). At 6 mpf, the ovaries of *egfra*+/− fish showed active folliculogenesis with all stages of follicles present; in contrast, the *egfra*−/− ovaries still remained translucent and their follicles remained arrested at PG stage. The cytoplasm of some mutant oocytes exhibited significant shrinkage generating large inter-follicular spaces, which were filled up by masses of stromal cells. At 9 mpf, the mutant ovaries continued to degenerate with much less follicles, and the stromal tissues continued to increase and spread, occupying most inter-follicular spaces. Interestingly, testicular tissues and spermatogenic cells started to arise among the stromal tissues of some females. At 12 mpf, the morphology of *egfra*-deficient gonads in females looked much like testis, which contained mostly spermatogenic cells undergoing active spermatogenesis with large amount of mature spermatozoa in the lumen. Remnants of follicles could still be seen but with amorphous structures undergoing disintegration. The ovaries of *egfra*+/− female remained normal with active folliculogenesis (Figure 9).

**FIGURE 10:**
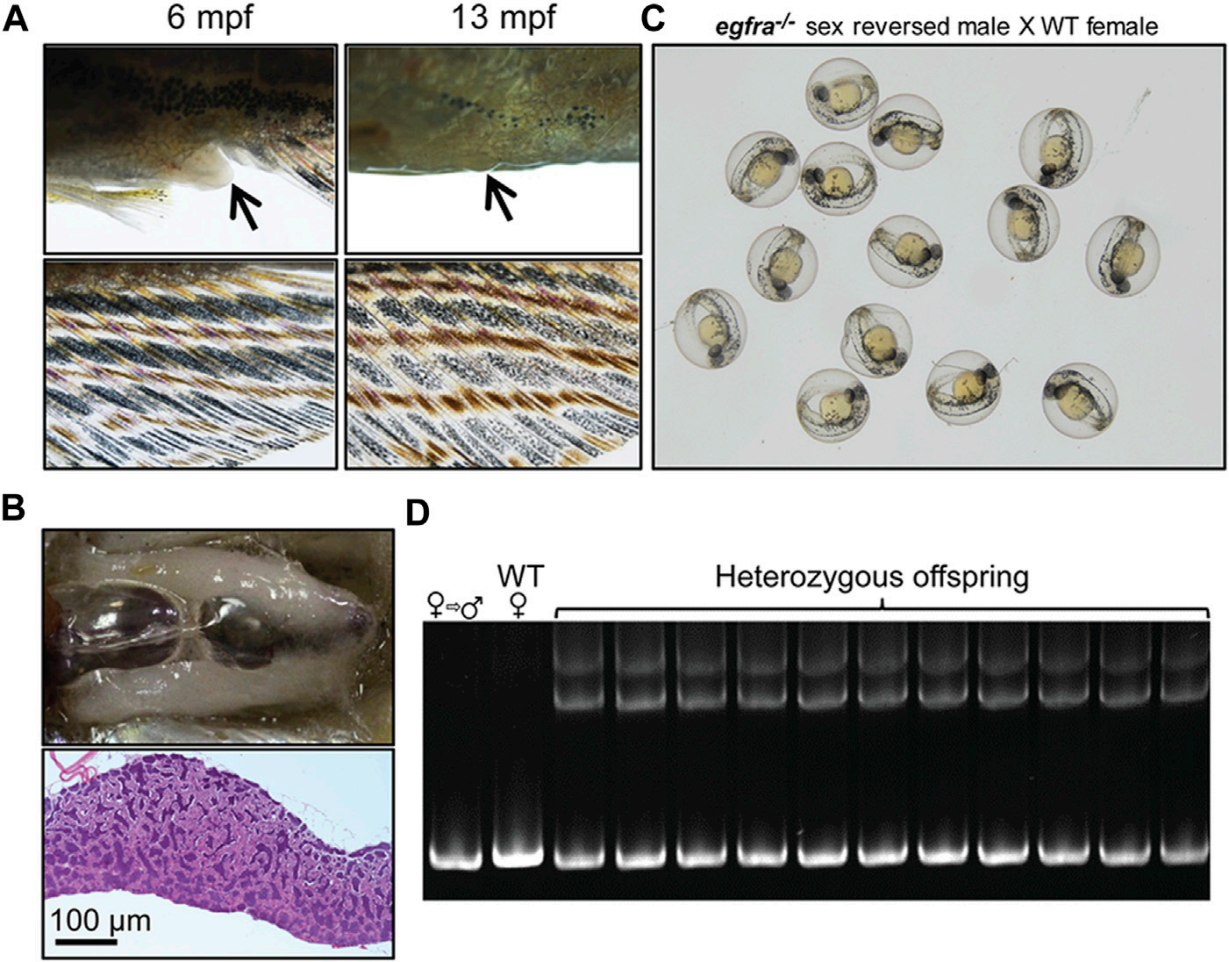
Development of secondary sexual characteristics during sex reversal and fertility test for sex-reversed males. **(A)** Protruding genital papilla (upper left, *arrow*) and a light anal fin (lower left) were observed in *egfra*−/− females at 6 mpf. The genital papilla disappeared and the anal fin turned golden in color in the same fish at 13 mpf. **(B)** The sex-reversed males (*egfra* ^−/−^) showed normal testis and spermatogenesis at 13 mpf. **(C)** The sex-reversed males were fertile and could spawn with WT females to produce normal embryos. **(D)** All the offspring of sex-reversed males and WT females were heterozygous *egfra*+/− as genotyped by HMA.

To further investigate the fertility of the sex-reversed males, we tested these fish by crossing them with WT females. The mutant *egfra*−/− females displaying feminine secondary sexual characteristics, such as protruding genital papilla and light anal fin, were selected out at 6 mpf and raised separately until 13 mpf when all these fish displayed masculine characteristics, including disappearance of genital papilla and golden coloration of the anal fin (Figure 10A). Dissection and histological examination showed well-developed testis with normal spermatogenesis but no oocytes (Figure 10B) These sexually reversed males were able to spawn naturally with WT females to produce viable offspring (Figure 10C). Genotyping showed that the *egfra* alleles of all the offspring were heterozygous as expected (Figure 10D).

### Interaction with the activin-inhibin system

Our previous studies proposed that EGF promotion of oocyte maturation in zebrafish was likely mediated by activins in follicles (Pang and Ge, 2002), and that EGF significantly stimulated expression of activin subunits (*inhbaa*, *inhbab*, and *inhbb*) (Pang and Ge, 2002; Wang and Ge, 2004a; Tse and Ge, 2010; Chung and Ge, 2012) but suppressed that of activin binding protein follistatin (*fsta*) (Wang and Ge, 2004a) in cultured follicle cells. Our recent study showed that the loss of inhibin (*inha*), an antagonist of activin, advanced follicle development by promoting follicle activation or PG–PV transition, leading to precocious puberty (Lu et al., 2020). This is opposite to the disruption of *egfra*, which blocked PG–PV transition as reported in this study. This raises an interesting question about potential interactions between EGFR signaling and activin-inhibin system in folliculogenesis. To address this issue, we created *egfra* and *inha* double mutant (*egfra−/−*; *inha−/−*)*.*

**FIGURE 11:**
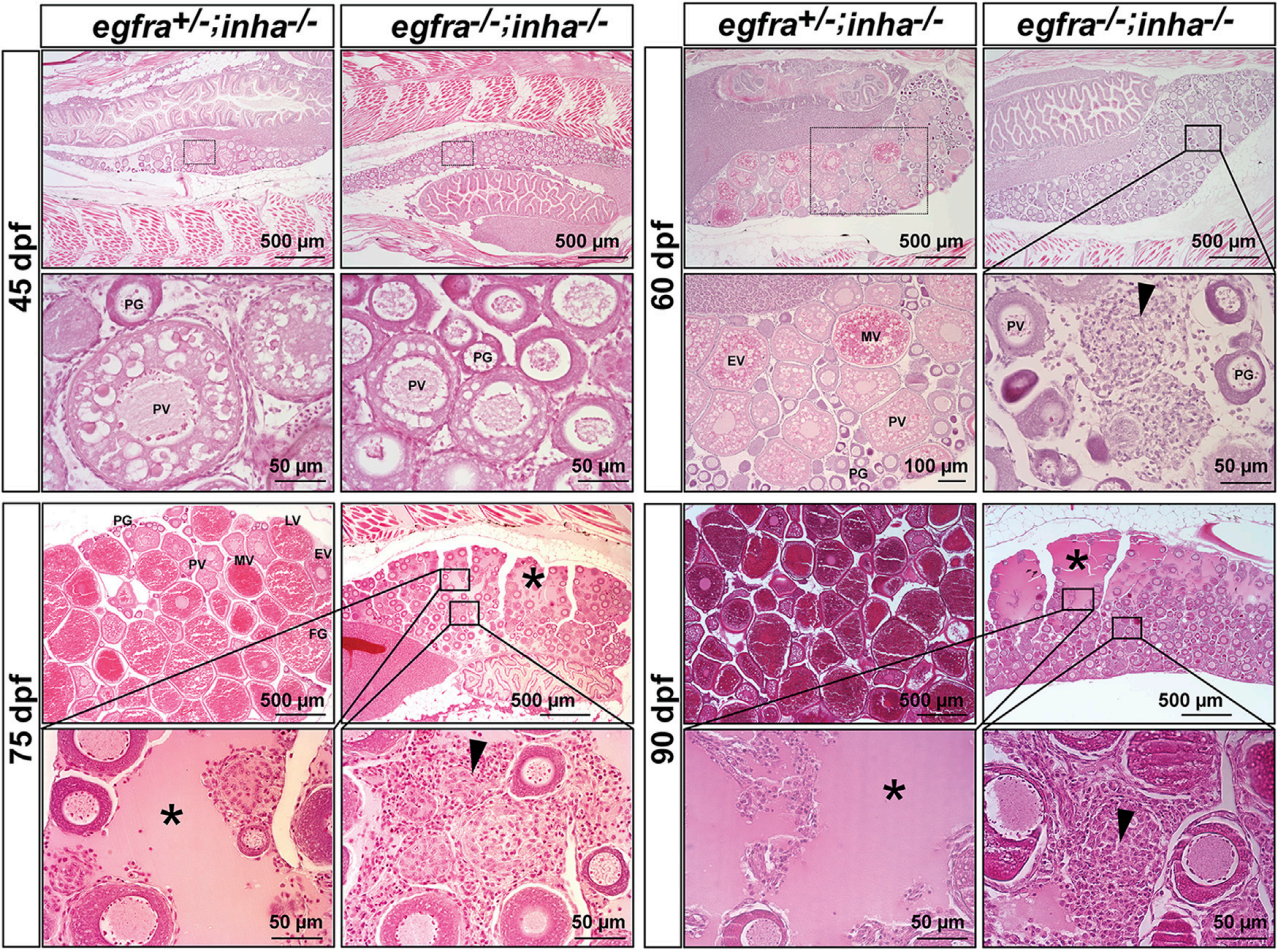
Double mutant of *egfra* and *inha* (*egfra* ^−/−^; *inha* ^−/−^) induced ovarian fibrosis and hydrovarium. The *egfra* ^+/−^ and *inha* ^−/-^ females were used as the control. Follicles were undergoing PG-PV transition at 45 dpf in both control and double mutant. These follicles continued to grow in the control ovary at 60 dpf with significant yolk accumulation in leading oocytes; however, the growth stopped completely at PG and early PV stages in the double mutant ovary (*egfra* ^−/−^; *inha* ^−/−^), which also showed fibrosis and accumulation of somatic stromal cells between follicles. The ovarian lamellae started to exhibit hydrovarium in the dorsal parts at 75 dpf in the double mutant, and the amount of fluid (asterisk) and fibrosis (arrowhead) continued to increase at 90 dpf. PG, primary growth; PV, previtellogenic; EV, early vitellogenic; MV, mid-vitellogenic; LV, late vitellogenic; FG, full-grown.

Using *egfra*+/−;*inha*−/− females as the control, we examined follicle growth and development in the double mutant at different time points of folliculogenesis. At 45 dpf, the leading follicles in the control had entered PV stage with multiple layers of cortical alveoli, marking the beginning of the secondary growth phase. In contrast, most follicles in the double mutant (*egfra−/−*; *inha−/−*) were at PG stage with only a few entering early PV stage as shown by the presence of a small number of cortical alveoli. The control ovary (*egfra+/−*; *inha−/−*) entered vitellogenic growth at 60 dpf with leading follicles accumulating abundant yolk granules. In contrast, the follicles in the ovary of double mutant remained unchanged without any advancement. Meanwhile, a substantial amount of stromal cells started to accumulate in the inter-follicular spaces, and the amount continued to increase at later stages (75, 90, and 150 dpf). Interestingly, some stromal cells developed concentrically around the places occupied or left by oocytes, suggesting possible origin of these cells from the follicle cells. Also interestingly, starting from 75 dpf, the dorsal part of ovarian lamellae became gradually devoid of follicles and the space was filled with serous fluid. This space continued to expand through 150 dpf with follicles being mostly located on the ventral side. Again, the follicles were arrested at PG stage without any advancement to late PV stage. The serous fluid remained in the ovarian lamellae without leaking into the ovarian cavity, and the follicles appeared to undergo liquefactive necrosis (Figure 11).

## Discussion

**FIGURE 12:**
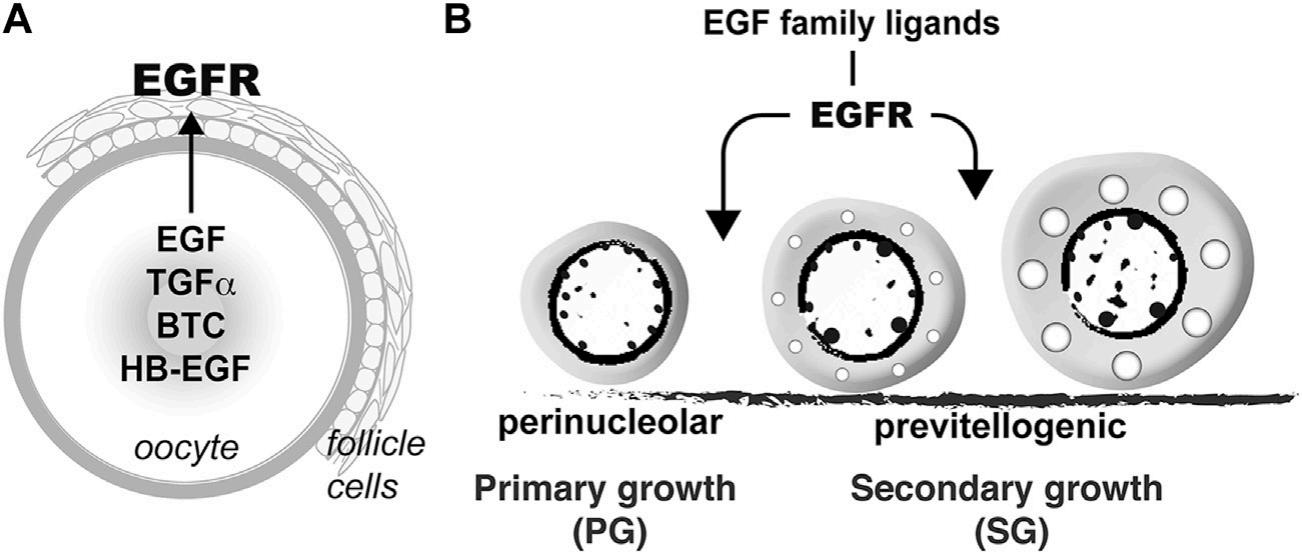
Hypothetical model on roles of EGFR signaling in controlling folliculogenesis in zebrafish. **(A)** Intrafollicular distribution of EGF family ligands and their receptor EGFR (Egfra) in the follicle (Tse and Ge, 2010), suggesting an oocyte-to-follicle cell signaling pathway. **(B)** EGF ligands-EGFR signaling pathway plays an important role in controlling early follicle development in zebrafish especially follicle activation or PG-PV transition.

Folliculogenesis is a dynamic process in the ovary, which is orchestrated by both endocrine and paracrine factors. The bidirectional communications between the oocyte and surrounding somatic follicle cells, the two main compartments of a follicle, constitute a pivotal paracrine signaling network in the ovary. In addition to providing metabolites to nourish the developing oocytes, the granulosa cells also release various paracrine factors that act on the oocytes (Monniaux, 2016). Meanwhile, instead of being passively regulated, the oocyte also plays active roles to regulate the surrounding somatic cells including the granulosa cells by releasing various growth factors (Erickson and Shimasaki, 2000), such as the well-characterized oocyte-specific growth differentiation factor 9 (GDF9) and bone morphogenetic protein 15 (BMP15) (Erickson and Shimasaki, 2001; Belli and Shimasaki, 2018; Roy et al., 2018). In zebrafish, our previous studies have revealed that in addition to GDF9/Gdf9 and BMP15/Bmp15, the oocyte may also signal the surrounding follicle cells *via* EGF–EGFR signaling pathway. The oocyte expresses a variety of EGF family ligands including EGF (Egf/*egf*), TGFα (Tgfa/*tgfa*), HB-EGF (Hbegf/*hbegf*) and BTC (Btc/*btc*) whereas their common receptor EGFR (Egfra/*egfra*) is exclusively expressed in the follicle cells, suggesting an intra-follicular paracrine signaling pathway from the oocyte to follicle cells in the zebrafish ovary (Wang and Ge, 2004a; Tse and Ge, 2010) (Figure 12A). This is further confirmed by a strong response of MAPK3/1 phosphorylation to EGF in both follicle layers of intact follicles and cultured follicle cells (Chung and Ge, 2012). One of the biological activities of EGF family ligands in zebrafish follicle cells is differential regulation of gonadotropin receptor (*fshr* and *lhcgr*) expression. EGF and its related peptides (TGFα, HB-EGF, and BTC) suppress *lhcgr* expression while promoting the expression of *fshr* in cultured follicle cells (Liu and Ge, 2013). In addition, EGF stimulates expression of all three activin/inhibin β subunits ((*inhbaa*, *inhbab*, and *inhbb*) in the follicle cells *via* different signaling pathways (Chung and Ge, 2012). Despite these studies, the functional importance of such intra-follicular signaling pathway remains largely unknown.

Using CRISPR/Cas9 method, we deleted the genes of *egf* and its receptor *egfra* in zebrafish. Surprisingly, although EGF is a well-documented growth factor involved in cell proliferation and differentiation in mammals (Carpenter and Cohen, 1990; Xian, 2007), the loss of Egf in zebrafish did not seem to cause any major abnormalities in somatic growth and reproduction. This result agrees well with that reported in EGF null mice, which showed no overt abnormalities in growth and organ development (Luetteke et al., 1999). The *egf* null zebrafish only exhibited minor reduction in growth rate over certain period (80–120 dpf) and reduced fertility in females after 7 mpf. Since *egf* is predominantly expressed in zebrafish gonads and its production site is primarily the oocytes in the ovary, the reduced fertilization rate of eggs from mutant females indicates a role for oocyte-derived Egf in long-term maintenance of quality egg production.

Considering that EGF family includes multiple ligands that share the same receptor and zebrafish oocytes express most of these ligands (Tse and Ge, 2010), we hypothesize that the lost functions of Egf might be compensated by other family members. To test this hypothesis, we went on to delete the gene of Egfra/*egfra*, the common receptor shared by EGF family, from the zebrafish genome. Surprisingly again, the loss of Egfra did not produce any significant defects in somatic growth except a slight reduction in growth rate, similar to that of *egf* mutant. This contrasts sharply with the targeted disruption of EGFR in mice, which resulted in embryonic or postnatal lethality, depending on genetic background of the mouse strains (Threadgill et al., 1995). However, the loss of Egfra in zebrafish had a profound effect on the fertility of females, but not males. While the male mutant showed normal fertility and spermatogenesis, the female mutant was infertile. Histological examination showed that Egfra null females had normal ovarian formation and differentiation; however, follicle development was completely arrested at PG or early PV stage with only a few oocytes containing small cortical alveoli, indicating a blockade at PG–PV transition or follicle activation. The functional importance of EGFR in mammalian ovary has also been demonstrated by genetic evidence in mice. While global knockout of EGFR in mice led to embryonic lethality, preventing further assessment of its role in reproduction, conditional knockout of EGFR in granulosa cells reduced oocyte maturation and ovulation, and therefore female fertility (Hsieh et al., 2011). It is interesting to note that although the loss of EGFR/Egfr impairs female fertility in both mouse and zebrafish, the defects occur at different time points of folliculogenesis. Disruption of EGFR signaling in zebrafish arrested follicle development at early stage, *viz*., follicle activation, whereas it impairs final stage of folliculogenesis, *viz*. final oocyte maturation, and ovulation, in mice. This difference could be due to global knockout in zebrafish vs. conditional knockout in granulosa cells in mice. It may also reflect different modes of actions of EGF family in the ovary of different species.

In mammals including humans, EGF is predominantly released from ovarian somatic cells and act as a paracrine factor working on the oocytes or granulosa cells, which express EGFR (Maruo et al., 1993; Bennett et al., 1996; Garnett et al., 2002; Conti et al., 2006). Interestingly, EGF-like factors in the follicle mediate LH signal in the final stage of folliculogenesis to induce oocyte maturation and ovulation (Park et al., 2004). In contrast to mammalian models, our previous studies in zebrafish showed that EGF family ligands in the ovary were mostly or exclusively expressed in the oocytes (Tse and Ge, 2010), while their receptor Egfra was exclusively expressed in the somatic follicle layer (Wang and Ge, 2004a; Tse and Ge, 2010) although there were also reports on the expression of *egfra* in the oocyte (Peyton and Thomas, 2011). This unique distribution of ligands and receptor in the follicle suggests that EGF family ligands may represent another class of oocyte-derived factors to mediate oocyte-to-follicle cell communications in zebrafish, in addition to GDF9 and BMP15. A similar mode of EGF family signaling has also been demonstrated in the ovary of other animals. In chicken ovary, EGF is secreted by oocytes at the germinal disc region (Volentine et al., 1998; Yao and Bahr, 2001b), and its receptor EGFR is expressed in the overlying granulosa cells (Yao and Bahr, 2001a). In quail ovary, EGFR is mostly expressed in the granulosa cells with weak expression in other cell types including oocytes, theca cells, and endothelial cells of blood vessels (Van Nassauw and Harrisson, 2000). In addition to EGF, chicken oocytes also express other EGF family members, such as HB-EGF, which acts as another potential oocyte-derived factor to regulate granulosa cell function (Wang et al., 2007). Further *in vitro* experiments showed that EGF and HB-EGF stimulated proliferation of chicken granulosa cells (Volentine et al., 1998; Yao and Bahr, 2001b; Wang et al., 2007) but suppressed their differentiation as evidenced by decreased expression of LH receptor (LHCGR) and progesterone production (Volentine et al., 1998; Yao and Bahr, 2001b). The inhibition of LHCGR expression in chicken ovary by EGF agrees well with our previous study in zebrafish that all four EGF family ligands tested (EGF, TGFα, HB-EGF, and BTC) suppressed basal and estrogen-stimulated Lhcgr/*lhcgr* expression in cultured follicle cells (Liu and Ge, 2013), suggesting a conserved function for EGF family ligands in vertebrate ovaries. Similar mechanism of EGFR-like signaling in the follicle has also been reported in insects. In *Drosophila*, the oocyte encodes a TGFα-like ligand *gurken* (*grk*), which signals through an EGFR-like receptor Torpedo/DER (*top/DER*) in the surrounding somatic cells (border cells) to guide their migration during oogenesis (Neuman-Silberberg and Schupbach, 1993; Duchek and Rorth, 2001; Ghiglione et al., 2002). All these studies suggest an oocyte-to-follicle cell paracrine signaling by EGFR and its ligands in ovarian follicles of both invertebrates and vertebrates.

The arrest of follicle development at PG-PV transition in *egfra* null mutant agrees well with the temporal expression profiles of EGF family ligands and *egfra* during folliculogenesis. The expression of both *egf* and *tgfa*, but not *hbegf* and *btc*, increased significantly at PG-PV transition, suggesting important roles for these oocyte-derived factors at this critical stage of development (Tse and Ge, 2010). Interestingly, *egfra* expression showed two major increases during folliculogenesis. The first one happened at PG–PV transition, corresponding to the increased expression of *egf* and *tgfa*, and the second one occurred dramatically at FG stage prior to oocyte maturation as shown in this study and reported previously (Tse and Ge, 2010), suggesting important roles for *egfra* at both early stage of follicle development, viz. follicle activation or PG-PV transition, and final stage of maturation and ovulation, which was similar to that reported in mammals (Park et al., 2004). Our data in this study fully support the importance of EGFR signaling in promoting PG-PV transition as follicle development in the *egfra* null female zebrafish was completely arrested at this stage with only a few follicles entering very early PV stage, which was marked by appearance of some small cortical alveoli (Figure 12B). However, EGF (Egf/*egf*) does not seem to be the major ligand responsible for Egfra signaling at this stage because *egf* null zebrafish showed normal follicle growth and maturation despite a decline in fertility at older age. There is a possibility that the loss of *egf* was compensated by other EGF family ligands from the oocyte (Tse and Ge, 2010). These EGF-like ligands may share redundant functions in regulating follicle development. Indeed, our previous study showed that both EGF and TGFα promoted zebrafish oocyte maturation (Pang and Ge, 2002). Unfortunately, we could not evaluate the importance of Egfra signaling at late stage of folliculogenesis, in particular oocyte maturation and ovulation, because all follicles were blocked at early stage.

To understand the molecular mechanisms underlying the defective follicle development induced by Egfra deficiency, we performed a transcriptome analysis on PG/PV follicles from the control and *egfra* mutant. The results showed an overall decline in metabolism, transcription, and cellular interaction in the absence of Egfra as genes involved in oxidative phosphorylation, oxidoreductase activity, cytomembrane transport, gene transcription, and cell adhesion were downregulated in *egfra*−/− ovary. Interestingly, several chemokine ligands in immune response pathway including *ccl25b*, *ccl34b.1*, *ccl34b.8*, and *ccl35.2* were all upregulated in the mutant. Since these chemokines are involved in positive regulation of ERK cascade, their upregulation of expression may explain the increased basal MAPK3/1 phosphorylation in *egfra* mutant ovary. Whether the abnormally higher basal level of MAPK3/1 phosphorylation is involved in generating *egfra* mutant phenotype remains unknown, and it would be an interesting issue to investigate in future studies. Transcriptome analysis also demonstrated decreased expression of *cyp19a1a* (ovarian aromatase) and *ar* (androgen receptor) in *egfra*−/− follicles, suggesting potential involvement of steroid signaling in early follicle development.

Interestingly, with folliculogenesis being blocked at PG/PV stage, all *egfra*−/− mutant females eventually changed to males through sex reversal at different time points of development. This is similar to the phenotype of follicle-stimulating hormone (FSH) receptor mutant (*fshr−/−*), *viz*., follicle arrest at PG stage followed by sex reversal of mutant females to males (Zhang et al., 2015a), and those of estrogen receptor mutants (Lu et al., 2017). In support of this was the increased expression of anti-Müllerian hormone (Amh/*amh*), a male-promoting gene, in *egfra*−/− follicle. This is again similar to that observed in *fshr*−/− mutant females (Zhang et al., 2015a).

One interesting observation was that several vitellogenin genes (*vtg1*, *vtg2*, *vtg4*, *vtg5*, *vtg6*, and *vtg7*) were expressed in the ovary at 45 dpf when PG-PV transition occurs and the loss of *egfra* (*egfra*−/−) resulted in a complete loss of expression of these genes in the mutant ovary. In fish, like other oviparous vertebrates, vitellogenin proteins are mostly produced by the liver in response to estrogen stimulation (Hara et al., 2016). Although vitellogenin expression in fish ovary has been reported in some studies (Wang et al., 2005; Zhong et al., 2014; Xue et al., 2018), its function remains entirely unknown. This would be an interesting issue to explore in future studies, especially its potential association with the failure of follicle activation in the *egfra*−/− mutant ovary.

Our previous studies have provided substantial evidence that activin-inhibin and EGF family ligands represent two major intra-follicular paracrine signaling pathways in zebrafish ovary. Activins are primarily produced by the follicle cells to act on the oocyte whereas EGF and its related peptides are produced mostly by the oocyte to regulate the follicle cells (Ge, 2005). EGF stimulated expression of all three activin subunits (*inhbaa*, *inhbab*, and *inhbb*) in cultured follicle cells *via* different signal transduction pathways (Chung and Ge, 2012), suggesting functional interaction between the two paracrine pathways. This was further evidenced in the present study by novel phenotypes shown by *egfra* and *inha* double mutant. Although the loss of inhibin (*inha−/−*) alone accelerated follicle activation or PG-PV transition (Lu et al., 2020), it could not rescue the phenotype of PG-PV blockade shown by the *egfra* mutant. Instead, *egfra* and *inha* double mutant induced a novel phenotype not shown by single mutants, *viz*. significant accumulation of serous fluid in ovarian lamellae. Although the underlying mechanism is unknown at this moment, it suggests that the activin-inhibin and EGFR signaling pathways are both implicated in controlling follicle development.

In contrast to *egfra*, the loss of *egfrb* caused no abnormal phenotypes in both growth and reproduction. Sequence analysis showed that Egfrb is a truncated protein showing high homology with the extracellular domain of Egfra. The existence of truncated forms of Egfra in zebrafish has been reported in our previous study. While cloning EGFR (Egfra) in zebrafish, we also isolated three shorter forms of cDNAs coding for the extracellular domain of EGFR, including EGFR8, 12 and 15 that correspond to exons 1–8, 1–12, and 1–15, respectively (Wang and Ge, 2004a). However, different from those truncated forms, which share identical nucleotide and amino acid sequences with Egfra and therefore may represent products of differential mRNA splicing, Egfrb is encoded by a different gene with significant sequence variation from Egfra at both nucleotide and amino acid levels. Since the loss of *egfrb* gene generated no obvious phenotype, the functional significance of Egfrb remains entirely unknown at this moment. Together with our previous report, the existence of truncated EGFR, either from differential splicing of the same mRNA or transcription of an independent gene, remains an interesting issue for further study in the future.

In conclusion, we performed genetic characterization of epidermal growth factor (Egf/*egf*) and its receptor (Egfra/*egfra*) in folliculogenesis and demonstrated an essential role for Egfra signaling in follicle activation or primary–secondary growth transition in zebrafish ovary (Figure 12). This, together with the data on temporospatial expression patterns of EGF ligands and Egfra in the ovary, strongly suggests a novel paracrine signaling pathway in the follicle that mediates oocyte-to-follicle cell communication. Our discovery once again points to the importance of oocyte in orchestrating folliculogenesis in vertebrates.

## Data Availability

The datasets presented in this study can be found in online repositories. The names of the repository/repositories and accession number(s) can be found below: https://www.ncbi.nlm.nih.gov/, SRR12432918-12432923.

## References

[B1] Belli M. Shimasaki S. (2018). Molecular Aspects and Clinical Relevance of GDF9 and BMP15 in Ovarian Function. Vitam Horm. 107, 317–348. 10.1016/bs.vh.2017.12.003 29544636 PMC6309678

[B2] Bennett R. A. Osathanondh R. Yeh J. (1996). Immunohistochemical Localization of Transforming Growth Factor-Alpha, Epidermal Growth Factor (EGF), and EGF Receptor in the Human Fetal Ovary. J. Clin. Endocrinol. Metab. 81 (8), 3073–3076. 10.1210/jcem.81.8.8768877 8768877

[B3] Carpenter G. Cohen S. (1990). Epidermal Growth Factor. J. Biol. Chem. 265 (14), 7709–7712. 10.1016/s0021-9258(19)38983-5 2186024

[B4] Chabot J.-G. St-Arnaud R. Walker P. Pelletier G. (1986). Distribution of Epidermal Growth Factor Receptors in the Rat Ovary. Mol. Cell Endocrinol. 44 (2), 99–108. 10.1016/0303-7207(86)90051-1 3005088

[B5] Chen B. Bronson R. T. Klaman L. D. Hampton T. G. Wang J.-f. Green P. J. (2000). Mice Mutant for Egfr and Shp2 Have Defective Cardiac Semilunar Valvulogenesis. Nat. Genet. 24 (3), 296–299. 10.1038/73528 10700187

[B6] Chen W. Ge W. (2013). Gonad Differentiation and Puberty Onset in the Zebrafish: Evidence for the Dependence of Puberty Onset on Body Growth but Not Age in Females. Mol. Reprod. Dev. 80 (5), 384–392. 10.1002/mrd.22172 23533185

[B7] Chung C.-K. Ge W. (2012). Epidermal Growth Factor Differentially Regulates Activin Subunits in the Zebrafish Ovarian Follicle Cells via Diverse Signaling Pathways. Mol. Cell Endocrinol. 361 (1-2), 133–142. 10.1016/j.mce.2012.03.022 22503865

[B8] Conti M. Hsieh M. Park J.-Y. Su Y.-Q. (2006). Role of the Epidermal Growth Factor Network in Ovarian Follicles. Mol. Endocrinol. 20 (4), 715–723. 10.1210/me.2005-0185 16051667

[B9] Dekel N. Sherizly I. (1985). Epidermal Growth Factor Induces Maturation of Rat Follicle-Enclosed Oocytes*. Endocrinology 116 (1), 406–409. 10.1210/endo-116-1-406 2578036

[B10] Ding J. Foxcroft G. R. (1994). Epidermal Growth Factor Enhances Oocyte Maturation in Pigs. Mol. Reprod. Dev. 39 (1), 30–40. 10.1002/mrd.1080390106 7999359

[B11] Downs S. M. (1989). Specificity of Epidermal Growth Factor Action on Maturation of the Murine Oocyte and Cumulus Oophorus *In Vitro*1. Biol. Reprod. 41 (2), 371–379. 10.1095/biolreprod41.2.371 2508778

[B12] Duchek P. Rørth P. (2001). Guidance of Cell Migration by EGF Receptor Signaling during *Drosophila* Oogenesis. Science 291 (5501), 131–133. 10.1126/science.291.5501.131 11141565

[B13] El-Hayek S. Demeestere I. Clarke H. J. (2014). Follicle-stimulating Hormone Regulates Expression and Activity of Epidermal Growth Factor Receptor in the Murine Ovarian Follicle. Proc. Natl. Acad. Sci. USA 111 (47), 16778–16783. 10.1073/pnas.1414648111 25385589 PMC4250110

[B14] Erickson G. F. Shimasaki S. (2001). The Physiology of Folliculogenesis: the Role of Novel Growth Factors. Fertil. Sterility 76 (5), 943–949. 10.1016/s0015-0282(01)02859-x 11704115

[B15] Erickson G. F. Shimasaki S. (2000). The Role of the Oocyte in Folliculogenesis. Trends Endocrinol. Metab. 11 (5), 193–198. 10.1016/s1043-2760(00)00249-6 10856922

[B16] Franks S. Hardy K. (2018). Androgen Action in the Ovary. Front. Endocrinol. 9, 452. 10.3389/fendo.2018.00452 PMC6097027 30147675

[B17] Garnett K. Wang J. Roy S. K. (2002). Spatiotemporal Expression of Epidermal Growth Factor Receptor Messenger RNA and Protein in the Hamster Ovary: Follicle Stage-specific Differential Modulation by Follicle-Stimulating Hormone, Luteinizing Hormone, Estradiol, and Progesterone1. Biol. Reprod. 67 (5), 1593–1604. 10.1095/biolreprod.102.005470 12390893

[B18] Ge W. (2005). Intrafollicular Paracrine Communication in the Zebrafish Ovary: the State of the Art of an Emerging Model for the Study of Vertebrate Folliculogenesis. Mol. Cell Endocrinol. 237 (1-2), 1–10. 10.1016/j.mce.2005.03.012 15921848

[B19] Ghiglione C. Bach E. A. Paraiso Y. Carraway K. L. 3rd Noselli S. Perrimon N. (2002). Mechanism of Activation of theDrosophilaEGF Receptor by the TGFα Ligand Gurken during Oogenesis. Development 129 (1), 175–186. 10.1242/dev.129.1.175 11782411

[B20] Hara A. Hiramatsu N. Fujita T. (2016). Vitellogenesis and Choriogenesis in Fishes. Fish. Sci. 82 (2), 187–202. 10.1007/s12562-015-0957-5

[B21] Harris R. Chung E. Coffey R. J. (2003). EGF Receptor Ligands. Exp. Cel Res. 284 (1), 2–13. 10.1016/s0014-4827(02)00105-2 12648462

[B22] Hernandez A. Bahr J. (2003). Role of FSH and Epidermal Growth Factor (EGF) in the Initiation of Steroidogenesis in Granulosa Cells Associated with Follicular Selection in Chicken Ovaries. Reproduction 125 (5), 683–691. 10.1530/rep.0.1250683 12713431

[B23] Hsieh M. Lee D. Panigone S. Horner K. Chen R. Theologis A. (2007). Luteinizing Hormone-dependent Activation of the Epidermal Growth Factor Network Is Essential for Ovulation. Mol. Cel Biol 27 (5), 1914–1924. 10.1128/MCB.01919-06 PMC1820474 17194751

[B24] Hsieh M. Thao K. Conti M. (2011). Genetic Dissection of Epidermal Growth Factor Receptor Signaling during Luteinizing Hormone-Induced Oocyte Maturation. PLoS One 6 (6), e21574. 10.1371/journal.pone.0021574 21738714 PMC3128061

[B25] Hsueh A. J. W. Welsh T. H. Jones P. B. C. (1981). Inhibition of Ovarian and Testicular Steroiodogenesis by Epidermal Growth Factor. Endocrinology 108, 2002–2004. 10.1210/endo-108-5-2002 6260473

[B26] Janz D. M. Van Der Kraak G. (1997). Suppression of Apoptosis by Gonadotropin, 17β-Estradiol, and Epidermal Growth Factor in Rainbow Trout Preovulatory Ovarian Follicles. Gen. Comp. Endocrinol. 105 (2), 186–193. 10.1006/gcen.1996.6820 9038251

[B27] Laird M. Thomson K. Fenwick M. Mora J. Franks S. Hardy K. (2017). Androgen Stimulates Growth of Mouse Preantral Follicles *In Vitro*: Interaction with Follicle-Stimulating Hormone and with Growth Factors of the TGFβ Superfamily. Endocrinology 158 (4), 920–935. 10.1210/en.2016-1538 28324051 PMC5460807

[B28] Lau E. S.-W. Zhang Z. Qin M. Ge W. (2016). Knockout of Zebrafish Ovarian Aromatase Gene (*Cyp19a1a*) by TALEN and CRISPR/Cas9 Leads to All-Male Offspring Due to Failed Ovarian Differentiation. Sci. Rep. 6, 37357. 10.1038/srep37357 27876832 PMC5120357

[B29] Liu K.-C. Ge W. (2013). Differential Regulation of Gonadotropin Receptors (*Fshr* and *Lhcgr*) by Epidermal Growth Factor (EGF) in the Zebrafish Ovary. Gen. Comp. Endocrinol. 181, 288–294. 10.1016/j.ygcen.2012.07.032 23036736

[B30] Lonergan P. Carolan C. Van Langendonckt A. Donnay I. Khatir H. Mermillod P. (1996). Role of Epidermal Growth Factor in Bovine Oocyte Maturation and Preimplantation Embryo Development *In Vitro*1. Biol. Reprod. 54 (6), 1420–1429. 10.1095/biolreprod54.6.1420 8724373

[B31] Lu H. Cui Y. Jiang L. Ge W. (2017). Functional Analysis of Nuclear Estrogen Receptors in Zebrafish Reproduction by Genome Editing Approach. Endocrinology 158 (7), 2292–2308. 10.1210/en.2017-00215 28398516

[B32] Lu H. Zhao C. Zhu B. Zhang Z. Ge W. (2020). Loss of Inhibin Advances Follicle Activation and Female Puberty Onset but Blocks Oocyte Maturation in Zebrafish. Endocrinology 161 (12), 1–19. 10.1210/endocr/bqaa184 33045050

[B33] Luetteke N. C. Qiu T. H. Fenton S. E. Troyer K. L. Riedel R. F. Chang A. (1999). Targeted Inactivation of the EGF and Amphiregulin Genes Reveals Distinct Roles for EGF Receptor Ligands in Mouse Mammary Gland Development. Development 126 (12), 2739–2750. 10.1242/dev.126.12.2739 10331984

[B34] Maack G. Segner H. (2003). Morphological Development of the Gonads in Zebrafish. J. Fish. Biol. 62, 895–906. 10.1046/j.1095-8649.2003.00074.x

[B35] Mann G. B. Fowler K. J. Gabriel A. Nice E. C. Williams R. L. Dunn A. R. (1993). Mice with a Null Mutation of the TGFα Gene Have Abnormal Skin Architecture, Wavy Hair, and Curly Whiskers and Often Develop Corneal Inflammation. Cell 73 (2), 249–261. 10.1016/0092-8674(93)90227-h 8477444

[B36] Maruo T. Ladines-Llave C. A. Samoto T. Matsuo H. Manalo A. S. Ito H. (1993). Expression of Epidermal Growth Factor and its Receptor in the Human Ovary during Follicular Growth and Regression. Endocrinology 132 (2), 924–931. 10.1210/endo.132.2.8425504 8425504

[B37] Miettinen P. J. Berger J. E. Meneses J. Phung Y. Pedersen R. A. Werb Z. (1995). Epithelial Immaturity and Multiorgan Failure in Mice Lacking Epidermal Growth Factor Receptor. Nature 376 (6538), 337–341. 10.1038/376337a0 7630400

[B38] Monniaux D. (2016). Driving Folliculogenesis by the Oocyte-Somatic Cell Dialog: Lessons from Genetic Models. Theriogenology 86 (1), 41–53. 10.1016/j.theriogenology.2016.04.017 27155734

[B39] Neuman-Silberberg F. S. Schüpbach T. (1993). The drosophila Dorsoventral Patterning Gene Gurken Produces a Dorsally Localized RNA and Encodes a TGFα-like Protein. Cell 75 (1), 165–174. 10.1016/s0092-8674(05)80093-5 7691414

[B40] Pang Y. Ge W. (2002). Epidermal Growth Factor and TGFα Promote Zebrafish Oocyte Maturationin Vitro: Potential Role of the Ovarian Activin Regulatory System. Endocrinology 143 (1), 47–54. 10.1210/endo.143.1.8579 11751590

[B41] Park J.-Y. Su Y.-Q. Ariga M. Law E. Jin S.-L. C. Conti M. (2004). EGF-like Growth Factors as Mediators of LH Action in the Ovulatory Follicle. Science 303 (5658), 682–684. 10.1126/science.1092463 14726596

[B42] Pati D. Balshaw K. Grinwich D. L. Hollenberg M. D. Habibi H. R. (1996). Epidermal Growth Factor Receptor Binding and Biological Activity in the Ovary of Goldfish, *Carassius auratus* . Am. J. Physiol. 270 (5 Pt 2), R1065–R1072. 10.1152/ajpregu.1996.270.5.R1065 8928907

[B43] Peyton C. Thomas P. (2011). Involvement of Epidermal Growth Factor Receptor Signaling in Estrogen Inhibition of Oocyte Maturation Mediated through the G Protein-Coupled Estrogen Receptor (Gper) in Zebrafish (*Danio rerio*)1. Biol. Reprod. 85 (1), 42–50. 10.1095/biolreprod.110.088765 21349822 PMC3123381

[B44] Qin M. Zhang Z. Song W. Wong Q. W.-L. Chen W. Shirgaonkar N. (2018). Roles of Figla/*figla* in Juvenile Ovary Development and Follicle Formation during Zebrafish Gonadogenesis. Endocrinology 159 (11), 3699–3722. 10.1210/en.2018-00648 30184072

[B45] Rieger D. Luciano A. M. Modina S. Pocar P. Lauria A. Gandolfi F. (1998). The Effects of Epidermal Growth Factor and Insulin-like Growth Factor I on the Metabolic Activity, Nuclear Maturation and Subsequent Development of Cattle Oocytes *In Vitro* . Reproduction 112 (1), 123–130. 10.1530/jrf.0.1120123 9538337

[B46] Roy S. Gandra D. Seger C. Biswas A. Kushnir V. A. Gleicher N. (2018). Oocyte-derived Factors (GDF9 and BMP15) and FSH Regulate AMH Expression via Modulation of H3K27AC in Granulosa Cells. Endocrinology 159 (9), 3433–3445. 10.1210/en.2018-00609 30060157 PMC6112599

[B47] Roy S. K. Greenwald G. S. (1990). Immunohistochemical Localization of Epidermal Growth Factor-like Activity in the Hamster Ovary with a Polyclonal Antibody*. Endocrinology 126 (3), 1309–1317. 10.1210/endo-126-3-1309 1689649

[B48] Schneider M. R. Wolf E. (2008). The Epidermal Growth Factor Receptor and its Ligands in Female Reproduction: Insights from Rodent Models. Cytokine Growth Factor. Rev. 19 (2), 173–181. 10.1016/j.cytogfr.2008.01.003 18295531

[B49] Sibilia M. Wagner E. F. (1995). Strain-dependent Epithelial Defects in Mice Lacking the EGF Receptor. Science 269 (5221), 234–238. 10.1126/science.7618085 7618085

[B50] Smitz J. Cortvrindt R. Hu Y. (1998). Epidermal Growth Factor Combined with Recombinant Human Chorionic Gonadotrophin Improves Meiotic Progression in Mouse Follicle-Enclosed Oocyte Culture. Hum. Reprod. 13 (3), 664–669. 10.1093/humrep/13.3.664 9572431

[B51] Srivastava R. K. Van Der Kraak G. (1995). Multifactorial Regulation of DNA Synthesis in Goldfish Ovarian Follicles. Gen. Comp. Endocrinol. 100 (3), 397–403. 10.1006/gcen.1995.1170 8775066

[B52] Threadgill D. W. Dlugosz A. A. Hansen L. A. Tennenbaum T. Lichti U. Yee D. (1995). Targeted Disruption of Mouse EGF Receptor: Effect of Genetic Background on Mutant Phenotype. Science 269 (5221), 230–234. 10.1126/science.7618084 7618084

[B53] Tse A. C.-K. Ge W. (2010). Spatial Localization of EGF Family Ligands and Receptors in the Zebrafish Ovarian Follicle and Their Expression Profiles during Folliculogenesis. Gen. Comp. Endocrinol. 167 (3), 397–407. 10.1016/j.ygcen.2009.09.012 19799903

[B54] Van Nassauw L. Harrisson F. (2000). Localisation of Epidermal Growth Factor Receptor in the Quail Ovary. Eur. J. Morphol. 38 (3), 145–152. 10.1076/0924-3860(200007)38:3;1-5;ft145 10916168

[B55] Volentine K. K. Yao H. H.-C. Bahr J. M. (1998). Epidermal Growth Factor in the Germinal Disc and its Potential Role in Follicular Development in the Chicken1. Biol. Reprod. 59 (3), 522–526. 10.1095/biolreprod59.3.522 9716549

[B56] Wang H. Tan J. T. T. Emelyanov A. Korzh V. Gong Z. (2005). Hepatic and Extrahepatic Expression of Vitellogenin Genes in the Zebrafish, *Danio rerio* . Gene 356, 91–100. 10.1016/j.gene.2005.03.041 15979250

[B57] Wang Y. Ge W. (2004a). Cloning of Epidermal Growth Factor (EGF) and EGF Receptor from the Zebrafish Ovary: Evidence for EGF as a Potential Paracrine Factor from the Oocyte to Regulate Activin/Follistatin System in the Follicle Cells1. Biol. Reprod. 71 (3), 749–760. 10.1095/biolreprod.104.028399 15115721

[B58] Wang Y. Ge W. (2004b). Developmental Profiles of Activin βA, βB, and Follistatin Expression in the Zebrafish Ovary: Evidence for Their Differential Roles during Sexual Maturation and Ovulatory Cycle1. Biol. Reprod. 71 (6), 2056–2064. 10.1095/biolreprod.104.032649 15329331

[B59] Wang Y. Li J. Ying Wang C. Yan Kwok A. H. Leung F. C. (2007). Epidermal Growth Factor (EGF) Receptor Ligands in the Chicken Ovary: I. Evidence for Heparin-Binding EGF-like Growth Factor (HB-EGF) as a Potential Oocyte-Derived Signal to Control Granulosa Cell Proliferation and HB-EGF and Kit Ligand Expression. Endocrinology 148 (7), 3426–3440. 10.1210/en.2006-1383 17395697

[B60] Xian C. J. (2007). Roles of Epidermal Growth Factor Family in the Regulation of Postnatal Somatic Growth. Endocr. Rev. 28 (3), 284–296. 10.1210/er.2006-0049 17322455

[B61] Xue R. Wang X. Xu S. Liu Y. Feng C. Zhao C. (2018). Expression Profile and Localization of Vitellogenin mRNA and Protein during Ovarian Development in Turbot (*Scophthalmus maximus*). Comp. Biochem. Physiol. B: Biochem. Mol. Biol. 226, 53–63. 10.1016/j.cbpb.2018.08.002 30114525

[B62] Yao H. H. C. Bahr J. M. (2001a). Chicken Granulosa Cells Show Differential Expression of Epidermal Growth Factor (EGF) and Luteinizing Hormone (LH) Receptor Messenger RNA and Differential Responsiveness to EGF and LH Dependent upon Location of Granulosa Cells to the Germinal Disc1. Biol. Reprod. 64 (6), 1790–1796. 10.1095/biolreprod64.6.1790 11369610

[B63] Yao H. H. C. Bahr J. M. (2001b). Germinal Disc-Derived Epidermal Growth Factor: A Paracrine Factor to Stimulate Proliferation of Granulosa Cells1. Biol. Reprod. 64 (1), 390–395. 10.1095/biolreprod64.1.390 11133698

[B64] Yoshimura Y. Tamura T. (1988). Effects of Gonadotrophins, Steroid Hormones, and Epidermal Growth Factor on the *In Vitro* Proliferation of Chicken Granulosa Cells. Poult. Sci. 67 (5), 814–818. 10.3382/ps.0670814 3136450

[B65] Zhang Z. Lau S.-W. Zhang L. Ge W. (2015a). Disruption of Zebrafish Follicle-Stimulating Hormone Receptor (*Fshr*) but Not Luteinizing Hormone Receptor (*Lhcgr*) Gene by TALEN Leads to Failed Follicle Activation in Females Followed by Sexual Reversal to Males. Endocrinology 156 (10), 3747–3762. 10.1210/en.2015-1039 25993524

[B66] Zhang Z. Zhu B. Ge W. (2015b). Genetic Analysis of Zebrafish Gonadotropin (FSH and LH) Functions by TALEN-Mediated Gene Disruption. Mol. Endocrinol. 29 (1), 76–98. 10.1210/me.2014-1256 25396299 PMC5414772

[B67] Zhong L. Yuan L. Rao Y. Li Z. Zhang X. Liao T. (2014). Distribution of Vitellogenin in Zebrafish (*Danio rerio*) Tissues for Biomarker Analysis. Aquat. Toxicol. 149, 1–7. 10.1016/j.aquatox.2014.01.022 24549118

[B68] Zhou R. Tsang A. H. K. Lau S.-W. Ge W. (2011). Pituitary Adenylate Cyclase-Activating Polypeptide (PACAP) and its Receptors in the Zebrafish Ovary: Evidence for Potentially Dual Roles of PACAP in Controlling Final Oocyte Maturation. Biol. Reprod. 85 (3), 615–625. 10.1095/biolreprod.111.091884 21636738

