# Supplementary material for: Disruption of Epidermal Growth Factor Receptor but Not EGF Blocks Follicle Activation in Zebrafish Ovary
Source: Front Cell Dev Biol. 2022 Jan 17;9:750888. doi: 10.3389/fcell.2021.750888 (PMC8802807; doi:10.3389/fcell.2021.750888)
Supplement: Supplementary file 2 [file Table1.DOCX]

**Table S1.** Sequences of primers used in this study.

| **Gene** | **Forward (5`-3`)** | **Product size (bp)** | **Accession number** |
| --- | --- | --- | --- |
| **Gene expression analysis** | | | |
| *egf*-Forward | GACTGCGATGTAAACGCTGA | 174 | NM 205622.2 |
| *egf*-Reverse | GCCATTTTTGTGTTGCAATG |  |  |
| *egfra*-Forward | AACGCAAATAATGGCAGGAC | 191 | AY657019.1 |
| *egfra*-Reverse | TCTCCAGAACCACAGTGCAG |  |  |
| *egfrb*-Forward | TGGGTTTGCGTTCACTGAAG | 203 | NM_205622.2 |
| *egfrb*-Reverse | ACAGCCTTCATCTCCACACA |  |  |
| *ef1a*-Forward | GATGCACCACGAGTCTCTGA | 158 | NM_131263 |
| *ef1a*-Reverse | TGATGACCTGAGCGTTGAAG |  |  |
|  |  |  |  |
| **Genotyping** | | | |
| *egfΔ2*-F | GGGGTCGACAAGAGAATGTCA | 108 | BX510944 |
| *egfΔ2*-R | CGGGTGTAAAAGGAGTTTCCA |  |  |
| *egfraΔ4*-F | GCCTGTAAGGACTTCCAGGATGAA | 189 | CR788232 |
| *egfraΔ4*-R | CCCTTGTAGAAACTCTGGAACAGCA |  |  |
| *egfΔ44*-F | CAATGAATGTTCCCTATGG | 133 | BX510944 |
| *egfΔ44*-R | TTCTTCAGTCCTACCTTG |  |  |
| *egfrb*-F | GTATGCGCTGTTGCTCAGAT | 125 | Unmapped |
| *egfrb*-R | TCTGGGTTTGCGTTCACTGA |  |  |
|  |  |  |  |
| **RT-PCR detection of mutant mRNA** | | | |
| *egf*-F1 | TCCCCTGCTGATGTTAAAGTG |  |  |
| *egf*-R1 | AGTGGCAGCGCAGTGTTTT |  |  |
| *egf*-F2 | AGTGGTGCATCCCATCAATCA |  |  |
| *egf*-F3 | GGGCTCTTACTTCTGCACCT |  |  |
| *egfra*-F3 | ATGCACTGGACCCAGACCTA |  |  |
| *egfra*-R3 | CCACAGCTGAATCATCAGGTA |  |  |
| *egfra*-F4 | TCTACGACCCAAACACACAC |  |  |
| *egfrb*-F2 | TCAAATCCCTCAGTGCCTTT |  |  |
| *egfrb*-R2 | ATTAGGGCCTTGCACTTTGT |  |  |
| *egfrb*-F3 | ATCAGTGACGGAGATGTGGT |  |  |
