# Supplementary figures and images for: Disruption of Epidermal Growth Factor Receptor but Not EGF Blocks Follicle Activation in Zebrafish Ovary
Source: Front Cell Dev Biol. 2022 Jan 17;9:750888. doi: 10.3389/fcell.2021.750888 (PMC8802807; doi:10.3389/fcell.2021.750888)

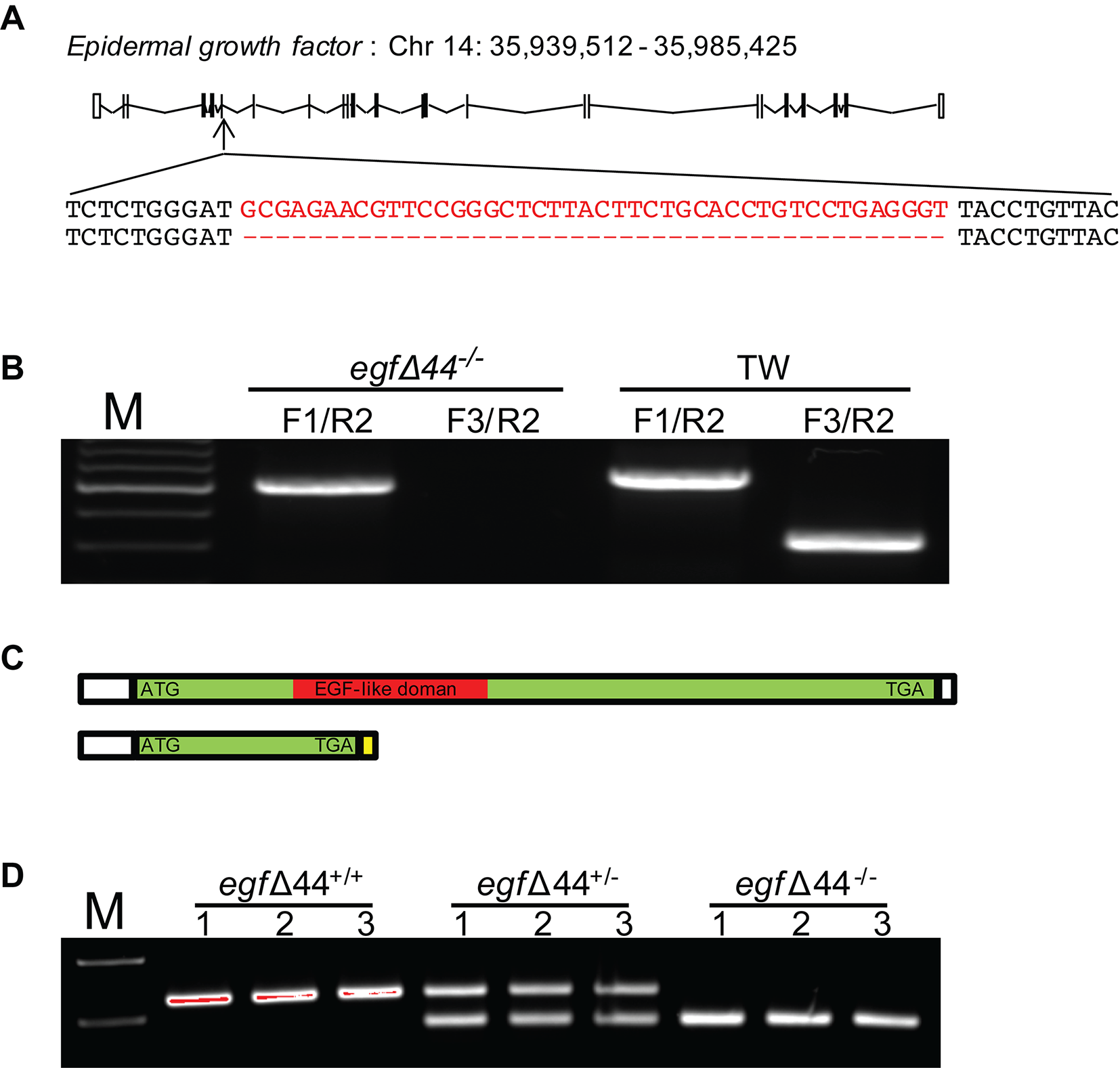

Supplement: Supplementary file 3 [file Image3.TIF]

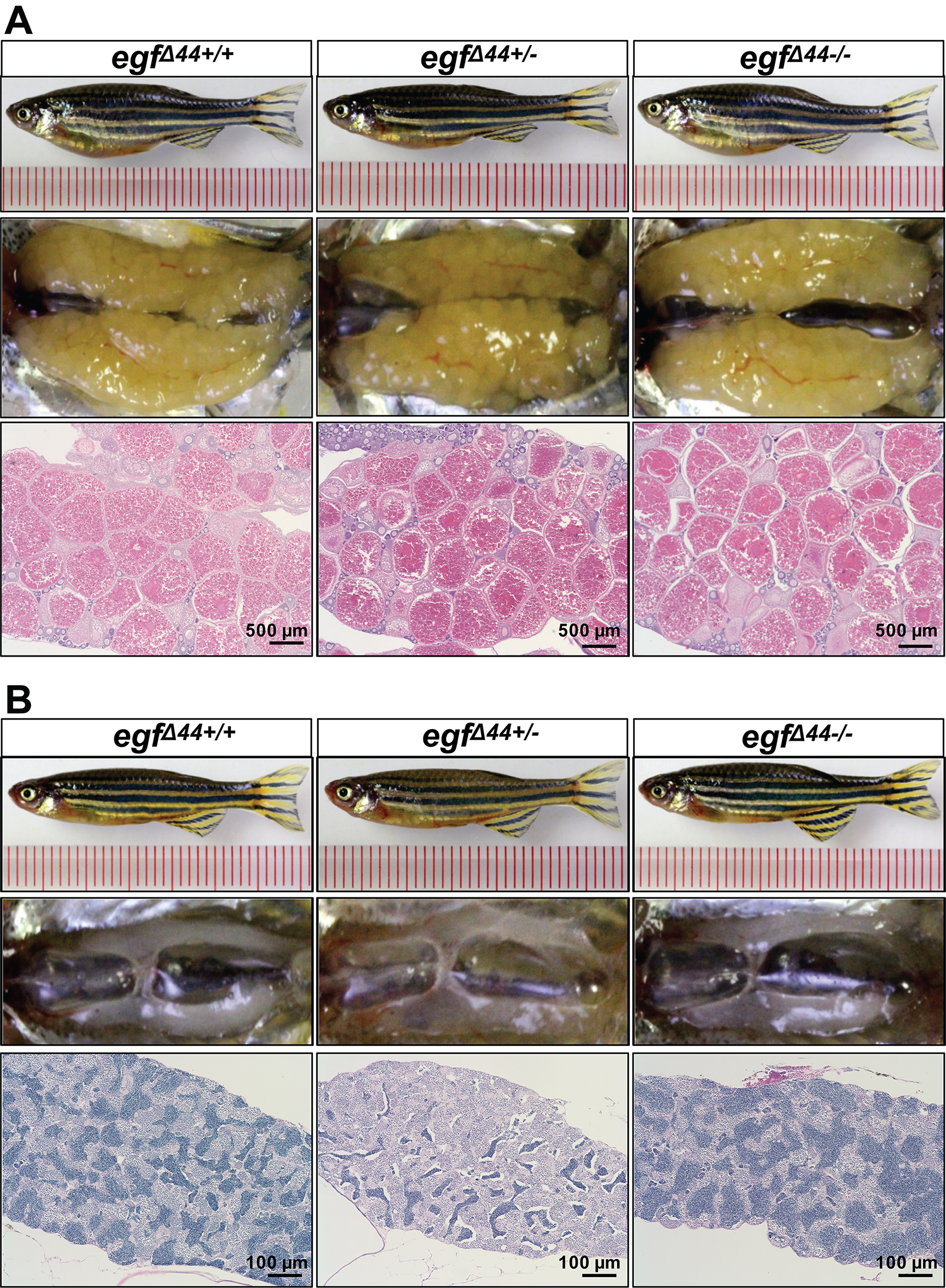

Supplement: Supplementary file 4 [file Image4.TIF]

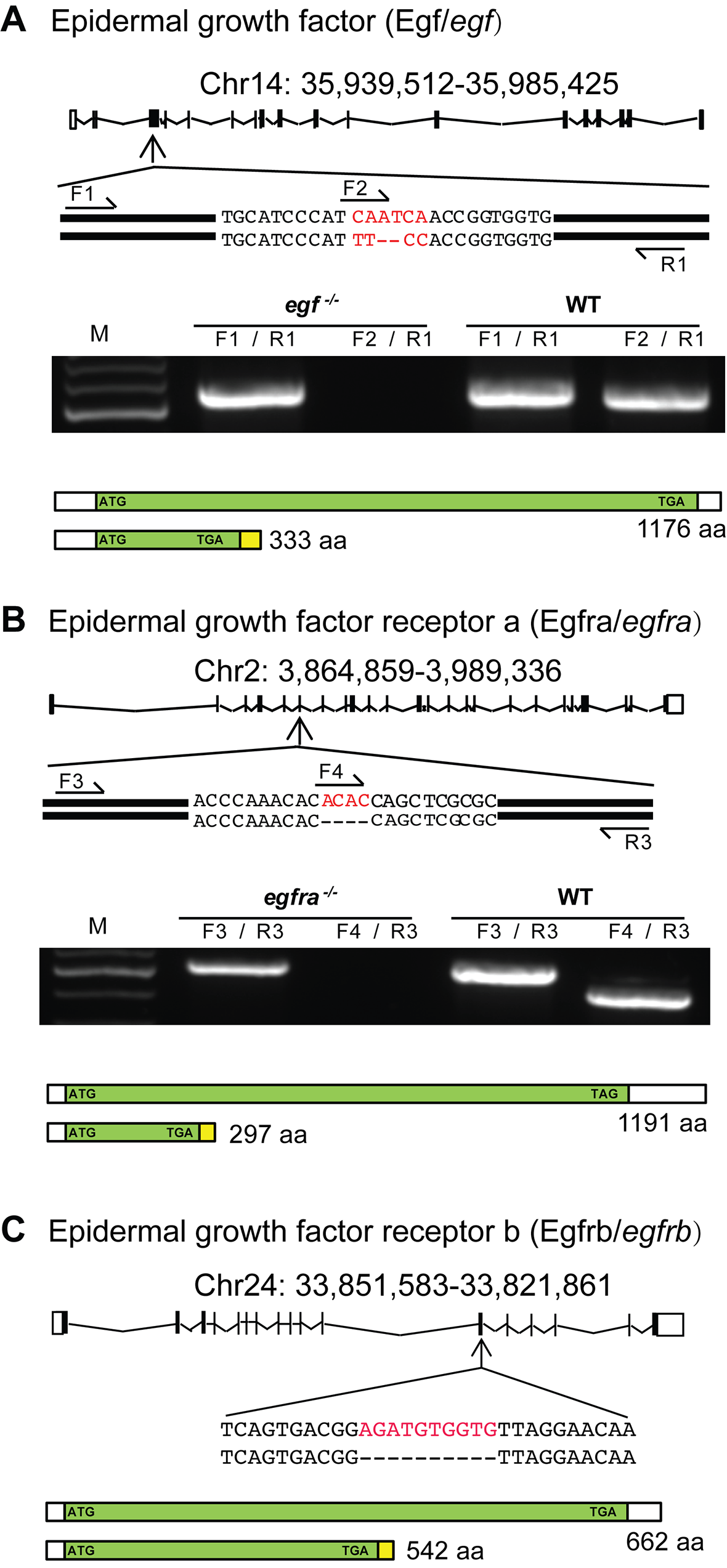

Supplement: Supplementary file 5 [file Image2.TIF]

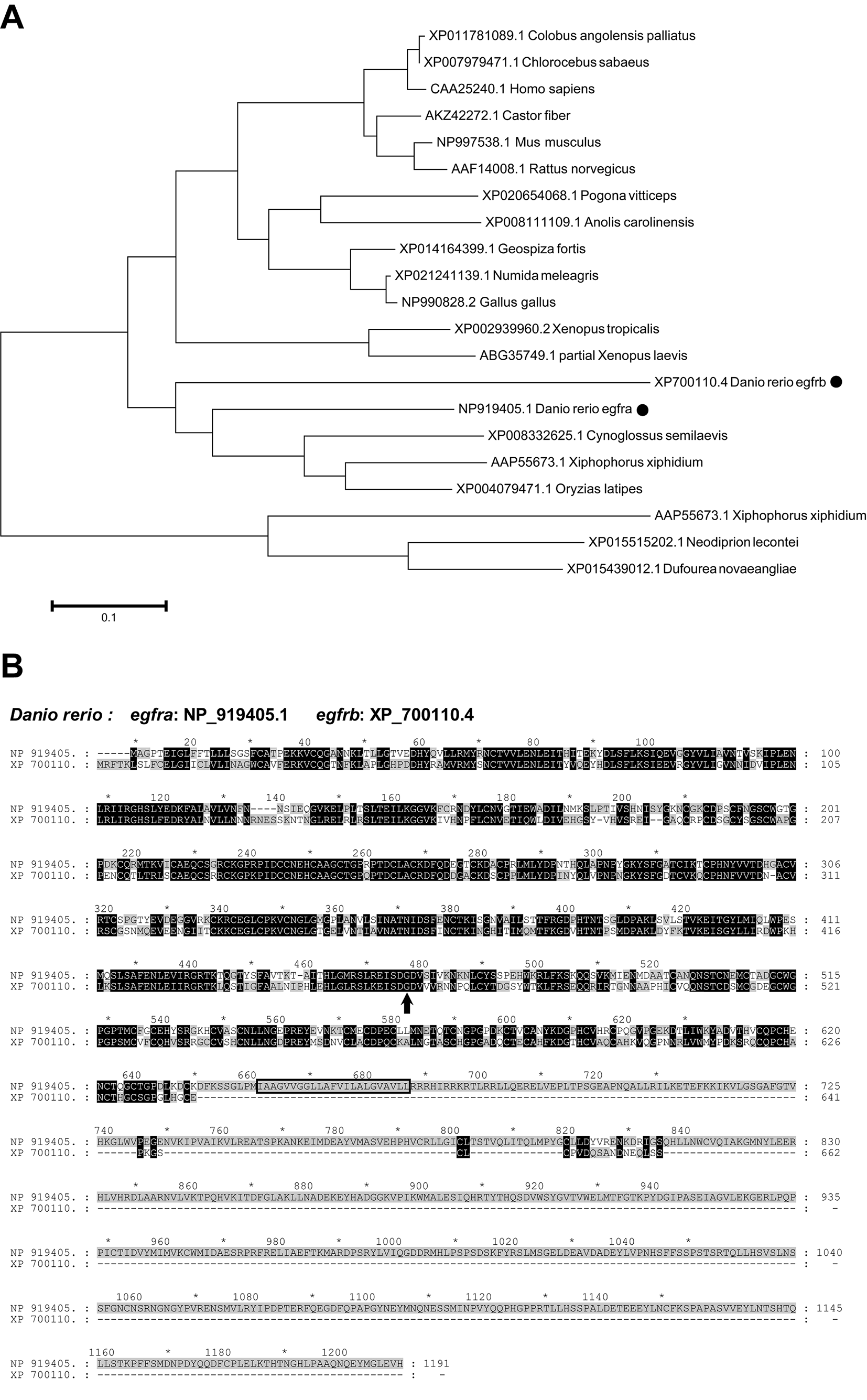

Supplement: Supplementary file 6 [file Image1.TIF]

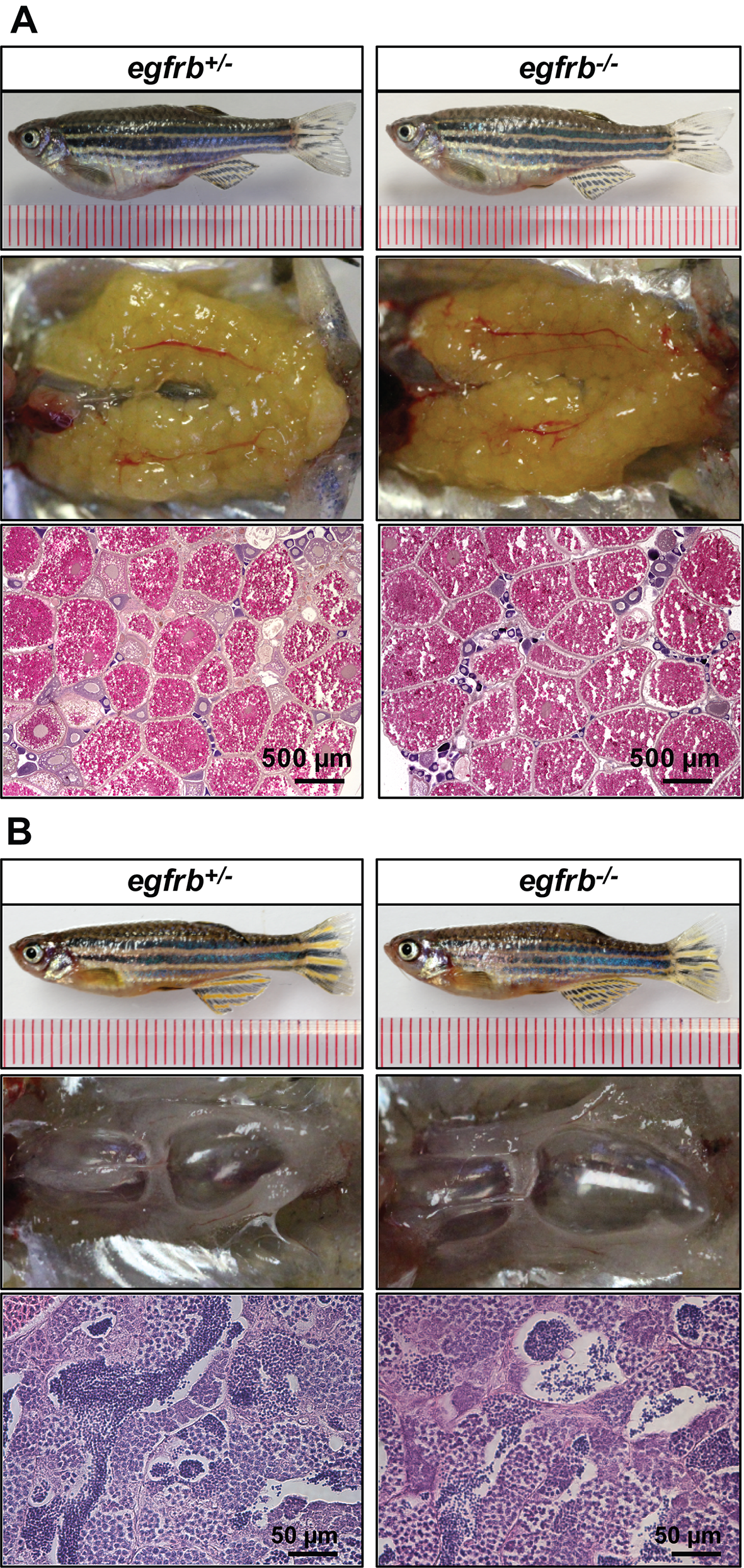

Supplement: Supplementary file 7 [file Image5.TIF]
