# Supplementary material for: Disruption of Epidermal Growth Factor Receptor but Not EGF Blocks Follicle Activation in Zebrafish Ovary
Source: Front Cell Dev Biol. 2022 Jan 17;9:750888. doi: 10.3389/fcell.2021.750888 (PMC8802807; doi:10.3389/fcell.2021.750888)
Supplement: Supplementary file 8 [file DataSheet1.DOCX]

**Supplemental Figure Legends**

**Fig. S1** (A) Phylogenetic analysis of vertebrate epidermal growth factor receptors showed that zebrafish Egfrb is located on a separate branch apart from zebrafish Egfra. The tree was constructed with the neighbor-joining method based on the amino acid sequences. (2) Amino acid sequence alignment of Egfra and Egfrb in zebrafish. The predicted Egfrb protein has 662 amino acid, and Egfra has 1191 amino acid. Egfrb showed high conservation with the N-terminal of Egfra. Arrow indicates the position of gRNA target sit in Egfrb.

**Fig. S2** Schematic representation of the genomic structures and generation of *egf,* *egfra* and *egfrb* mutants using CRISPR/Cas9 technique. (A) A 6-bp deletion and 4-bp insertion were introduced at the target site resulting in a net 2-bp deletion in *egf* gene. RT-PCR analysis showed no signal in the mutant ovary with mutation-specific primers (F2/R1). (B) A 4-bp deletion was introduced in *egfra* gene, and RT-PCR on the ovary showed no detection of signal with mutation-specific primers (F4/R3). (C) A 10-bp deletion was introduced in *egfrb* gene. All mutations generated frame shifting and introduced early stop codons in *egf,* *egfra* and *egfrb* mRNAs to produce truncated proteins.

**Fig. S3** Schematic representation of the genomic structure of *egf* gene and generation of *egf*^Δ44-/-^ mutant using CRISPR/Cas9 technique. (A) A 44-bp deletion was introduced in the coding sequence. (B) Demonstration of the mutant transcript in the ovary by RT-PCR with mutant-specific primer F3. (C) The frameshifting deletion introduced an early stop codon in *egf* generating a truncated protein missing the EGF-like domain. (D) Genotyping analysis of *egf*^Δ44+/+^, *egf*^Δ44+/-^, and *egf*^Δ44-/-^ zebrafish.

**Fig. S4** Gonadal development of *egf*^Δ44-/-^ mutant. (A) Anatomical and histological examination of the ovary in *egf*^Δ44-/-^ mutant and controls (*egf*^Δ44+/+^, *egf*^Δ44+/-^). The mutant follicles developed normally. (B) Anatomical and histological examination of the testis in *egf*^Δ44-/-^ mutant and controls (*egf*^Δ44+/+^, *egf*^Δ44+/-^). The spermiogenesis was normal in *egf*-deficient males.

**Fig. S5** Gonadal development of *egfrb* mutant. (A) Anatomical and histological examination of the ovary in *egfrb* mutant (*egfrb*-/-) and the control (*egfrb*+/-). The mutant ovary developed normally with normal folliculogenesis. (B) Anatomical and histological examination of the testis in *egfrb* mutant (*egfrb*-/-) and the control (*egfrb*+/-). The spermiogenesis was normal in *egfrb*-deficient males.
